# Multicenter development of an interpretable machine learning model for prognosis of acute pancreatitis with acute kidney injury

**DOI:** 10.3389/fmed.2026.1955191

**Published:** 2026-10-29

**Authors:** Wei Wang, Tongping Shen

**Affiliations:** 1 The First Affiliated Hospital of Anhui University of Chinese Medicine, Hefei, China; 2 School of Information Engineering, Anhui University of Chinese Medicine, Hefei, China

**Keywords:** acute kidney injury, acute pancreatitis, clinical prognostic factors, machine learning, shiny application, survival prediction

## Abstract

**Background:**

Patients with severe acute pancreatitis complicated by acute kidney injury (AP-AKI) face extremely high mortality rates; therefore, early and precise risk stratification is crucial for optimizing individualized treatment strategies. However, traditional linear prognostic models have inherent limitations in capturing the non-linear associations between complex clinical features and patient outcomes, and they often lack sufficient clinical generalizability. This study aimed to develop, externally validate, and deploy a highly accurate and interpretable machine learning model to predict the survival outcome of AP-AKI patients.

**Methods:**

Early clinical data of AP-AKI patients were extracted from the large intensive care database MIMIC-IV, with all-cause mortality at 60 days serving as the primary observation endpoint. The study utilized three algorithms (Boruta, LASSO, and MRMR) to screen 18 core features, subsequently constructing and systematically comparing 24 machine learning models. The models underwent comprehensive evaluation through the Area Under the Receiver Operating Characteristic Curve (AUC), calibration metrics, and Decision Curve Analysis (DCA), while external validation was accomplished in the independent eICU database. Additionally, the SHAP method was applied to deeply clarify the complex non-linear interaction patterns among prognostic factors, and a convenient interactive online application was developed based on the optimal model.

**Results:**

Among the 24 included models, the Random Forest (RF) model demonstrated the best holistic performance. It achieved an AUC of 0.8963 (0.8479, 0.9447) in the internal validation set. Despite facing extreme class imbalance and cross-center data heterogeneity in the eICU external validation set, the model maintained excellent discrimination with an AUC of 0.8122 (0.7139, 0.9106). Compared with numerous traditional and machine learning baseline models, the RF model exhibited optimal overall calibration, and DCA confirmed that it achieved highly valuable clinical net benefit across a wide range of decision thresholds. SHAP analysis profoundly revealed multiple non-linear driving patterns, such as step-wise or “S-shaped” threshold effects of the 18 core features (prominently including aki_kdigo_stage, charlson_comorbidity_index, rdw, ast, and sapsii_score) on mortality risk. The successfully deployed interactive Shiny web application (https://medicalpredictor.shinyapps.io/Online_Prediction_of_60-Day_Survival_Status_in_Patients) enables code-free individualized risk prediction and feature attribution interpretation.

**Conclusions:**

This study successfully constructed and externally validated a highly interpretable RF model for the precise prediction of survival outcomes in AP-AKI patients. The online interactive application deployed based on this model provides a convenient, intuitive, and evidence-based decision support tool for early clinical risk stratification and individualized precision intervention. Future multi-center, prospective randomized controlled studies are urgently needed to further validate its real-world clinical utility, along with exploring the inclusion of time-series data to develop more comprehensive longitudinal dynamic predictive models.

## Introduction

Acute pancreatitis (AP) is an inflammatory disease of the pancreas with a spectrum of severity ranging from mild pancreatitis to severe acute pancreatitis (SAP) (1). It is caused by multiple factors, including gallstones, alcohol consumption, hypertriglyceridemia, and medications (2). AP presents with clinical manifestations varying from mild abdominal pain to multiple organ dysfunction syndrome (3). In 2021, an estimated 5.9 million people worldwide were living with pancreatitis, 2.7 million new cases were diagnosed, and 122,000 deaths were attributed to the disease (4).

Acute kidney injury (AKI) is one of the most common and severe complications of SAP, with a reported incidence ranging from 14% to 42% (5). Studies have shown that pancreatitis complicated with AKI significantly increases mortality (6–8). The pathogenesis of AKI in SAP is multifactorial, involving hemodynamic changes, inflammatory mediators, oxidative stress, and direct nephrotoxicity (9). Therefore, early identification and management of AKI in SAP patients are crucial for improving prognosis and reducing mortality (10).

As an important branch of artificial intelligence, machine learning (ML) can uncover complex nonlinear correlations between features and disease outcomes. It has been widely applied in disease diagnosis, complication monitoring, and prognosis prediction, providing auxiliary support for clinical decision-making (11–16). Notably, numerous studies have utilized ML techniques to construct prediction tools for AP, with a primary focus on predicting disease severity, complications, and mortality (17–21).

However, most existing studies are limited to retrospective, single-center designs, with drawbacks such as small sample sizes and limited model interpretability. To address these gaps, this study aims to develop and validate an interpretable machine learning model to predict survival in patients with AP complicated by AKI. The model employs the Shapley Additive exPlanations (SHAP) method to elucidate the impact of key features and their potential interactions. Additionally, a Shiny-based interactive web application has been developed to improve the model's clinical applicability.

## Methods

### Study design and study population

Data for this study were sourced from the Medical Information Mart for Intensive Care IV (MIMIC-IV version 3.1) and the eICU Collaborative Research Database (v2.0) (22, 23). The author, Tongping Shen, completed the mandatory training required by the National Institutes of Health (NIH) and successfully obtained access to these databases (Certification Number: 14348115). As all database data were fully anonymized and de-identified, the requirement for written informed consent from participants was waived. This study was conducted and reported in strict accordance with the Strengthening the Reporting of Observational Studies in Epidemiology (STROBE) guidelines.

The inclusion criteria for this study were as follows: (1) Confirmed diagnosis of acute pancreatitis based on International Classification of Diseases (ICD) codes; (2) Concurrent clinical diagnosis of acute kidney injury; (3) Age >= 18 years; (4) Follow-up duration exceeding 60 days, or a clear record of a 60-day mortality outcome. The exclusion criteria were: (1) Patients with AKI who had pre-existing end-stage kidney disease (ESKD); (2) Hospital length of stay less than 24 h; (3) Cases presenting extreme physiological outliers, data exceeding reasonable clinical ranges, or logical errors.

The definitions of all core clinical indicators in this study strictly adhered to international standard guidelines. The severity of acute pancreatitis was classified according to the 2012 revised Atlanta classification (24): mild acute pancreatitis was defined by the absence of organ failure and local/systemic complications; moderately severe acute pancreatitis was defined by the presence of transient organ failure (duration < 48 h) or concurrent local/systemic complications; SAP was defined by the presence of persistent organ failure (duration > 48 h). Organ failure was established using the modified Marshall scoring system or the Sequential Organ Failure Assessment (SOFA) score, defined as a score of >= 2 for any organ system.

The diagnosis of AKI was based on the 2012 kidney disease: Improving Global Outcomes (KDIGO) guidelines (25). Specific diagnostic indicators were extracted utilizing the dynamic calculation table (mimiciv_derived.kdigo_stages) officially provided by MIMIC-IV. The criteria included: an absolute increase in serum creatinine (SCr) by >= 26.5 umol/L (0.3 mg/dL) within 48 h, or a relative increase of >= 50% from baseline (where the dynamic baseline creatinine is defined as the lowest value recorded within the 7 days preceding each assessment point); or a weight-adjusted urine output of < 0.5 mL/(kg·h) sustained for more than 6 h. To ensure the chronological logic of disease progression, AP-AKI patients included in this study were strictly limited to those with new-onset AKI within 7 days following AP diagnosis and ICU admission (26). Concurrently, to comprehensively evaluate the overall impact of kidney injury on prognosis, we extracted the maximum stage recorded throughout the entire ICU stay (aki_kdigo_stage) to serve as a core baseline severity feature for the machine learning predictive models.

In the MIMIC-IV database, 60-day mortality was determined by the date of death (dod), accurately capturing both in-hospital and out-of-hospital deaths. Because eICU lacks post-discharge follow-up, we applied highly stringent length-of-stay criteria to ensure outcome accuracy: mortality was defined exclusively for patients who died in the hospital within 60 days; survival was defined exclusively for patients explicitly documented as hospitalized and alive at or beyond the 60-day mark (record offset >= 86,400 min). Patients discharged alive before 60 days were excluded.

Following the application of these inclusion and exclusion criteria, a final cohort of 721 subjects was enrolled. The patient screening flowchart is illustrated in Supplementary Figure 1. The 721 patients ultimately included represent a severe subgroup with concurrent AKI from the original acute pancreatitis cohort. A comparative analysis of the baseline characteristics between the included and excluded groups is detailed in Supplementary Table 1. All clinical data for the enrolled patients were obtained from routine clinical records.

### Data preprocessing and feature selection

In this study, the original dataset comprising 721 samples was partitioned into training and validation sets at a 7:3 ratio. For outlier exclusion, this study adopted a combined strategy using both statistical methods and clinical judgment. We first applied standard statistical rules to flag potential outliers. These flagged values were then evaluated against biologically plausible boundaries. To prevent the loss of genuine but extreme clinical data, we exclusively removed values that were both statistically anomalous and clinically impossible. Subsequently, for variables with a missing data rate of less than 20%, Multiple Imputation was employed to impute the missing values, thereby maximizing the retention of sample information. To resolve the class imbalance issue within the training set (where the sample sizes for the two outcomes were 394 and 112, respectively), the Synthetic Minority Over-sampling Technique (SMOTE) algorithm was employed for oversampling-based balancing, which equalized the sample size of both classes to 394 each. To strictly prevent data leakage, we performed the entire feature selection process using the Boruta, LASSO, and MRMR algorithms exclusively on the isolated training set. The test set was strictly held out and remained completely unseen during all feature engineering and model training procedures, serving solely for final model evaluation.

Concurrently, a series of quality control procedures were implemented, including outlier removal and the standardized encoding of categorical variables. These preprocessing steps established a high-quality data foundation for subsequent machine learning model construction; the preprocessing outcomes for the training set are illustrated in Figure 1.

**Figure 1:**
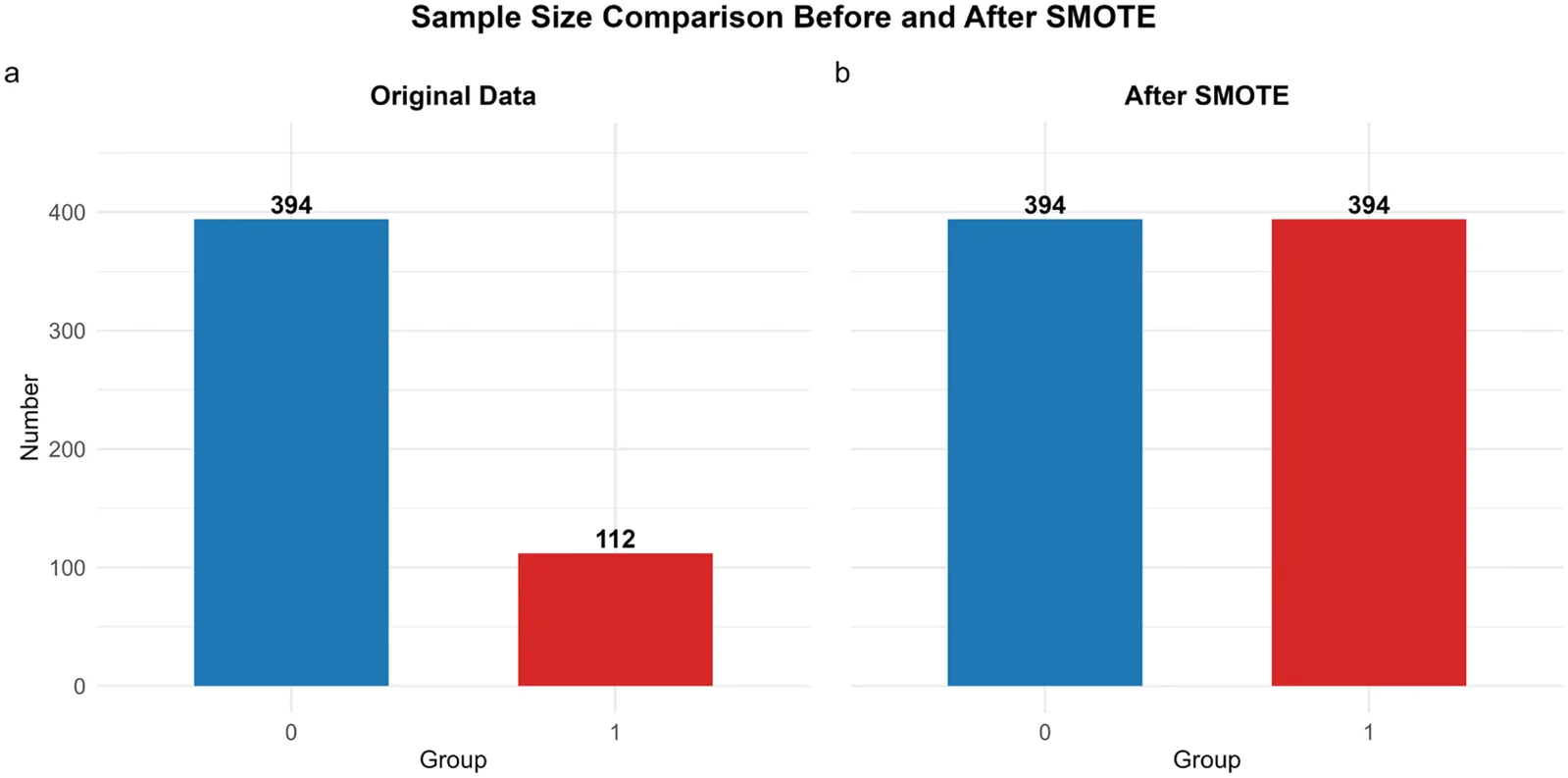
Comparison of training set sample size before and after SMOTE processing.

Three feature selection algorithms were employed in this study: the Boruta algorithm, the Least Absolute Shrinkage and Selection Operator (LASSO) algorithm, and the Maximal Relevance Minimal Redundancy (MRMR) algorithm. Cross-verification via these multiple algorithms was implemented to enhance the reliability of the selected features, and the overlapping distribution of the feature selection results is illustrated in the Venn diagram in Figure 2.

**Figure 2:**
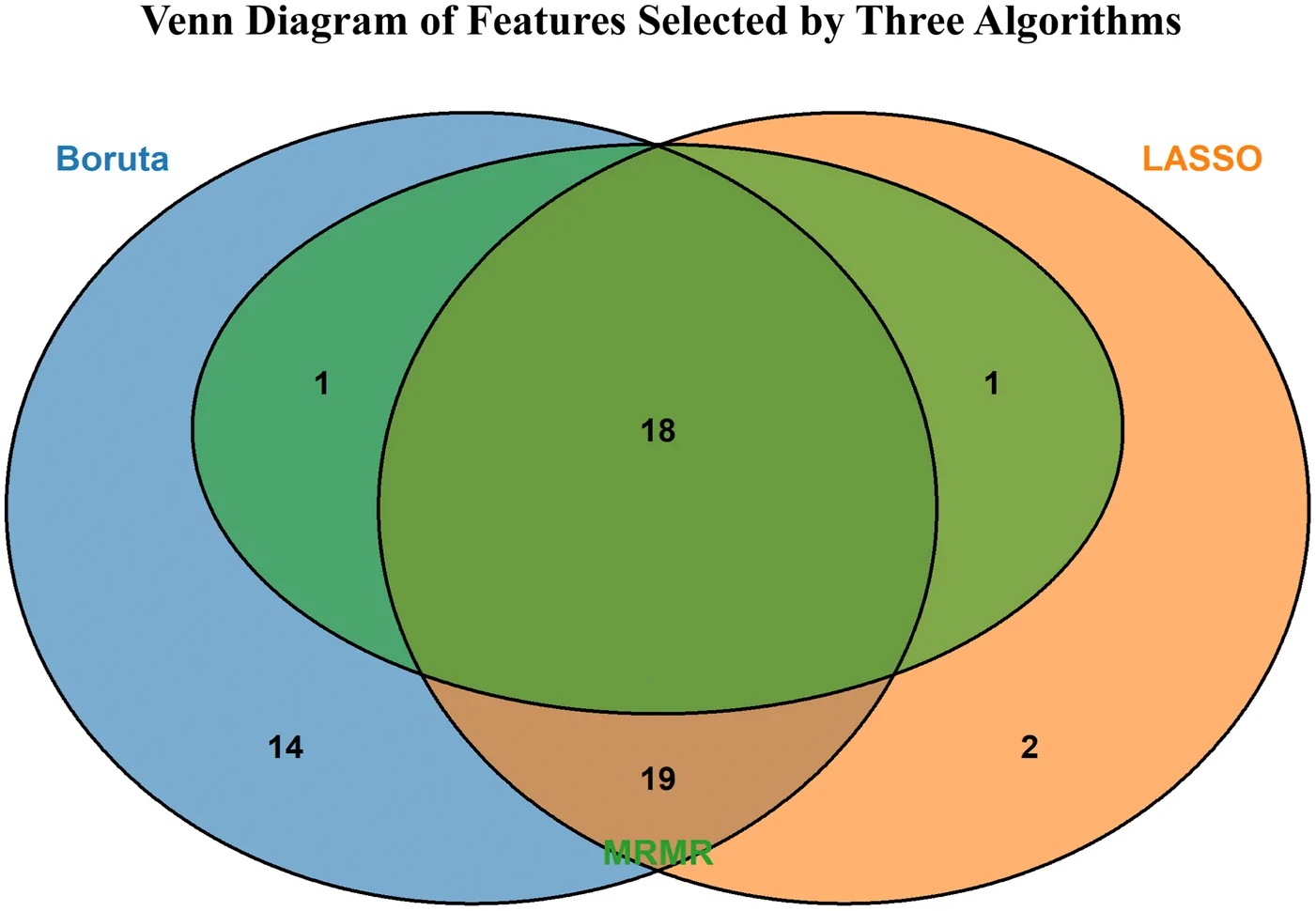
Venn diagram of features selected by three algorithms.

The Boruta algorithm is a random forest-based feature selection method that identifies valid predictors by comparing the importance of real features against randomly generated “shadow features.” Its core advantage lies in its ability to minimize feature selection bias and robustly identify variables with genuine predictive value.

The LASSO algorithm is a feature screening method designed for high-dimensional data based on $L_{1}\;$ regularization. $L_{1}\;$ penalty term on the regression coefficients, it shrinks the coefficients of redundant features to zero, thereby simultaneously achieving dimensionality reduction and model regularization. Its strengths include automated core feature selection, effective mitigation of overfitting risks, and excellent generalization performance.

The MRMR algorithm is an information theory-based feature selection method. It filters for an optimal feature subset characterized by both high predictive value and low redundancy by maximizing the relevance between features and the outcome variable, while minimizing the mutual redundancy among the features. Its primary advantage is the effective elimination of interference from redundant features, which substantially enhances the interpretability and generalization capabilities of the resulting feature subset.

### Model construction and validation

In this study, we selected 24 machine learning algorithms, including linear, tree-based, ensemble, and neural network models. The algorithm set specifically includes the C5.0 decision tree algorithm, Random Forest (RF), Ridge Regression (RIDGE), Support Vector Machine (SVM), Elastic Net, Partial Least Squares DA (PLSDA), Bayesian GLM, LASSO Regression, Generalized Linear Model, Linear Discriminant Analysis, Flexible Discriminant Analysis, LightGBM (LGBM), AdaBoost, eXtreme Gradient Boosting (XGB), Sparse Linear Discriminant Analysis (SLDA), Gradient Boosting Machine (GBM), Deep Neural Network, Bagging Decision Trees (BDT), Naive Bayes, Neural Network, Quadratic Discriminant Analysis (QDA), Conditional Inference Tree, K-Nearest Neighbors, and Classification and Regression Tree.

All machine learning models in this study were developed using the caret package in R. The models incorporated 18 clinical and laboratory features as input variables, with binary overall survival (OS) status serving as the outcome variable. To ensure model robustness, hyperparameter tuning was systematically conducted using a grid search strategy. This process was paired with 10-fold cross-validation for internal model optimization, targeting the AUC as the primary optimization metric. All models were configured to generate class probabilities to ensure consistency in evaluation, and parallel computing was enabled during training to enhance computational efficiency.

Statistical validation for model comparison was performed by combining the Bootstrap method and DeLong's test. Specifically, 1,000 Bootstrap resampling's with replacement were executed on the test set to calculate each model's accuracy, Kappa coefficient, sensitivity, specificity, positive/negative predictive values, precision, recall, F1-score, and AUC. The 95% confidence intervals (95% CIs) were constructed using the 2.5th and 97.5th percentiles of the Bootstrap estimates. To determine the statistical significance of differences between models, DeLong's test was utilized for pairwise comparisons of the AUCs. For the other classification performance metrics, significance was evaluated based on the overlap of their 95% CIs: non-overlapping 95% CIs indicated a statistically significant difference, whereas overlapping 95% CIs denoted no significant difference. All models were evaluated using an identical analytical pipeline to ensure the fairness and reliability of the multiple comparisons.

Calibration ability was visually illustrated by calibration curves that reflected the consistency between the model's predicted probabilities and actual clinical outcomes, and the integrated Brier score was further used to quantitatively evaluate calibration accuracy. Specifically, the integrated Brier score quantifies the deviation between the predicted survival probabilities and actual survival status, thereby comprehensively reflecting the model's calibration efficacy and fitting quality (27).

With respect to clinical utility, Decision Curve Analysis (DCA) was performed to assess the model’s clinical net benefit at different threshold probabilities, to verify its value in practical clinical decision-making scenarios.

### Model interpretation and deployment

An explanation method based on SHAP technology was adopted to analyze and discuss the model with the optimal overall performance, aiming to reveal its prediction mechanism and feature contributions (28).

To elucidate the model's prediction mechanism in depth, this study identified key predictive factors using summary and dependence plots from the SHAP method and explored their correlations with patients’ survival outcomes. Meanwhile, latent feature interactions were uncovered via SHAP interactive plots. To improve the model's usability and accessibility, the optimized predictive model was ultimately deployed as a web-based, interactive Shiny application that supports personalized survival prediction and customized interpretive explanations. As a lightweight web framework in the R language ecosystem, Shiny enables rapid packaging of R-built models and visualization results into interactive web applications without professional programming knowledge, allowing clinical staff without a technical background to directly operate and use the model through a web browser.

### Statistical analysis

Categorical variables were described using counts and percentages, while continuous variables were summarized as median (interquartile range, IQR) or mean ± standard deviation (SD), depending on their distribution characteristics. For comparisons of data distribution differences between groups, the chi-square (*χ*^2^) test was used to analyze differences in categorical variables, whereas the Mann–Whitney U test was employed to evaluate differences in continuous variables. All tests were two-tailed, and a P-value < 0.05 was considered statistically significant.

All statistical analyses and chart generation in this study were performed using R software (version 4.3.1). Core analytical tasks including the plotting of AUC curves and the construction and training of machine learning models were completed with the assistance of packages such as plotROC, caret, pROC, and e1071.

The overall architecture of the prediction model designed in this study is shown in Figure 3, which includes modules of data preprocessing, feature selection, model construction and validation.

**Figure 3:**
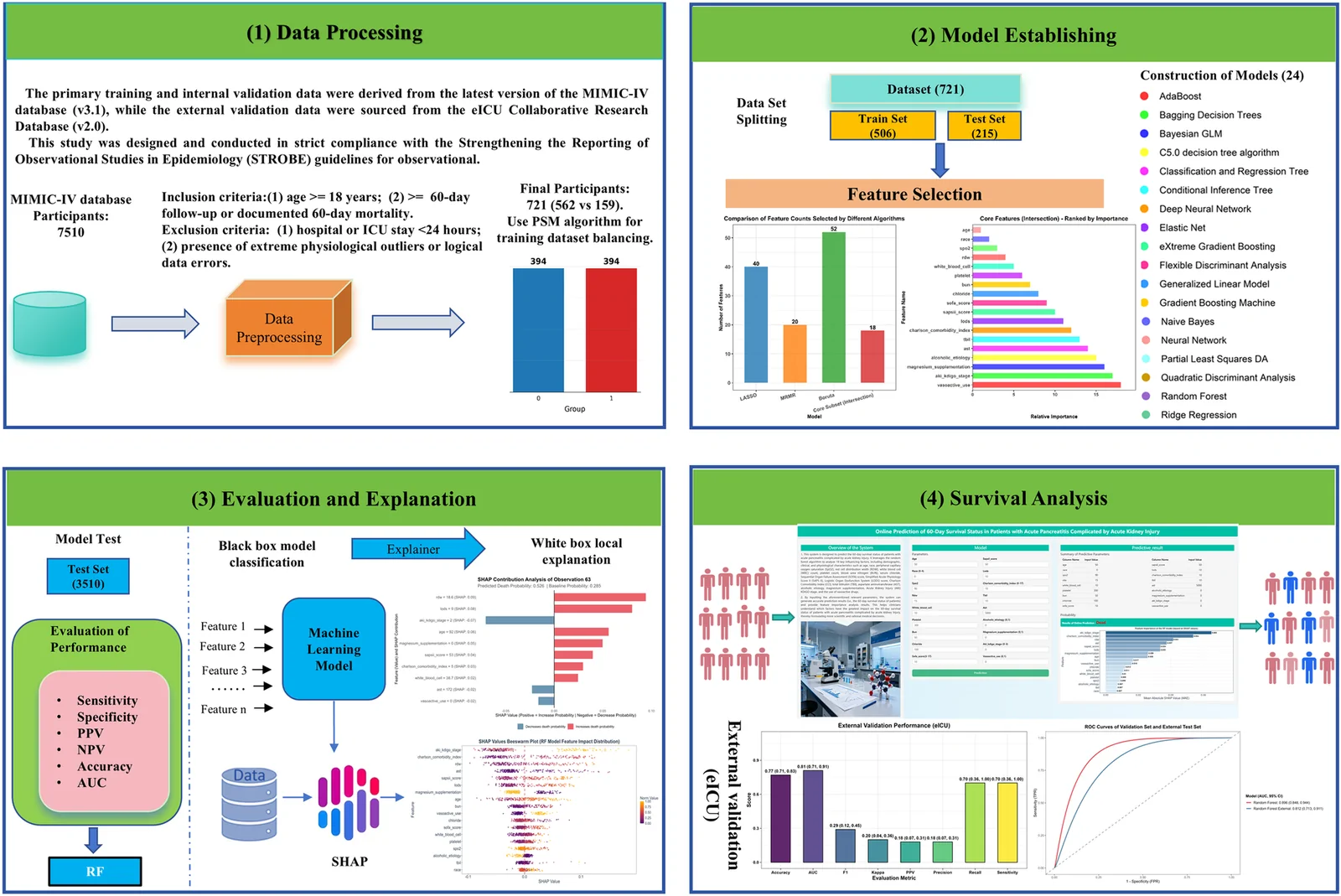
Framework diagram of the survival status prediction model.

## Results

### Patient characteristics

A total of 721 patients with acute pancreatitis complicated by AP-AKI were included in this retrospective analysis. Based on their survival outcomes, the study cohort was divided into a survivor group (562 cases) and a non-survivor group (159 cases). The detailed clinical characteristics of the study population are presented in Table 1.

**Table 1:** Baseline characteristics of patients with acute pancreatitis complicated by acute kidney injury at 60 days.

Characteristic	Statistic	Alive (*N* = 562)	Dead (*N* = 159)	OR (univariable)	OR (multivariable)
age	Mean ± SD	59.6 ± 18.0	70.4 ± 15.3	1.04 (1.03–1.05, *p* < .001)	1.03 (1.00–1.05, *p* = .020)
race	White	376 (66.9%)	103 (64.8%)		
	Black	47 (8.4%)	11 (6.9%)	0.85 (0.43–1.71, *p* = .656)	0.88 (0.35–2.25, *p* = .792)
	Asian	17 (3%)	6 (3.8%)	1.29 (0.50–3.35, *p* = .603)	1.46 (0.44–4.89, *p* = .539)
	Hispanic	24 (4.3%)	0 (0%)	0.00 (0.00-Inf, *p* = .975)	0.00 (0.00-Inf, *p* = .981)
	Other/Unknown	98 (17.4%)	39 (24.5%)	1.45 (0.94–2.23, *p* = .089)	1.81 (0.97–3.38, *p* = .064)
spo2	Mean ± SD	96.2 ± 2.1	95.6 ± 2.9	0.90 (0.84–0.97, *p* = .007)	0.89 (0.81–0.98, *p* = .022)
rdw	Mean ± SD	15.1 ± 2.0	16.8 ± 2.8	1.35 (1.25–1.46, *p* < .001)	1.33 (1.20–1.48, *p* < .001)
white_blood_cell	Mean ± SD	16.3 ± 8.8	19.0 ± 11.9	1.03 (1.01–1.04, *p* = .002)	1.01 (0.98–1.03, *p* = .587)
platelet	Mean ± SD	248.4 ± 138.9	241.0 ± 169.9	1.00 (1.00-1.00, *p* = .571)	
bun	Mean ± SD	27.0 ± 22.6	41.4 ± 28.6	1.02 (1.01–1.03, *p* < .001)	1.00 (0.99–1.01, *p* = .905)
chloride	Mean ± SD	106.2 ± 6.2	106.4 ± 8.2	1.00 (0.98–1.03, *p* = .768)	
sofa_score	0–3	221 (39.3%)	29 (18.2%)		
	4–7	219 (39%)	41 (25.8%)	1.43 (0.86–2.38, *p* = .173)	0.57 (0.28–1.17, *p* = .127)
	8–11	95 (16.9%)	52 (32.7%)	4.17 (2.49–6.97, *p* < .001)	0.97 (0.38–2.48, *p* = .945)
	>=12	27 (4.8%)	37 (23.3%)	10.44 (5.57–19.59, *p* < .001)	1.21 (0.34–4.36, *p* = .768)
sapsii_score	Mean ± SD	35.5 ± 14.3	52.1 ± 15.9	1.07 (1.06–1.08, *p* < .001)	1.00 (0.98–1.03, *p* = .807)
lods	0–3	205 (36.5%)	18 (11.3%)		
	4–6	211 (37.5%)	35 (22%)	1.89 (1.04–3.44, *p* = .038)	1.04 (0.48–2.27, *p* = .913)
	7–9	101 (18%)	53 (33.3%)	5.98 (3.33–10.73, *p* < .001)	2.33 (0.93–5.86, *p* = .072)
	>=10	45 (8%)	53 (33.3%)	13.41 (7.18–25.05, *p* < .001)	3.34 (1.00–11.11, *p* = .050)
charlson_comorbidity_index	0	67 (11.9%)	3 (1.9%)		
	1–2	165 (29.4%)	12 (7.5%)	1.62 (0.44–5.94, *p* = .463)	0.56 (0.12–2.75, *p* = .479)
	3–5	202 (35.9%)	54 (34%)	5.97 (1.81–19.72, *p* = .003)	2.32 (0.51–10.42, *p* = .274)
	>=6	128 (22.8%)	90 (56.6%)	15.70 (4.79–51.49, *p* < .001)	3.96 (0.83–18.93, *p* = .085)
tbil	Mean ± SD	2.4 ± 2.9	5.4 ± 7.9	1.13 (1.08–1.18, *p* < .001)	1.09 (1.04–1.15, *p* = .001)
ast	Mean ± SD	371.5 ± 1,334.1	1,450.6 ± 3,736.9	1.00 (1.00-1.00, *p* < .001)	1.00 (1.00-1.00, *p* = .019)
alcoholic_etiology	0	388 (69%)	134 (84.3%)		
	1	174 (31%)	25 (15.7%)	0.42 (0.26–0.66, *p* < .001)	0.83 (0.42–1.63, *p* = .582)
magnesium_supplementation	0	203 (36.1%)	82 (51.6%)		
	1	359 (63.9%)	77 (48.4%)	0.53 (0.37–0.76, *p* < .001)	0.41 (0.25–0.70, *p* < .001)
aki_kdigo_stage	1	155 (27.6%)	17 (10.7%)		
	2	271 (48.2%)	39 (24.5%)	1.31 (0.72–2.40, *p* = .377)	1.39 (0.66–2.92, *p* = .386)
	3	136 (24.2%)	103 (64.8%)	6.91 (3.94–12.12, *p* < .001)	4.02 (1.91–8.44, *p* < .001)
vasoactive_use	0	416 (74%)	94 (59.1%)		
	1	146 (26%)	65 (40.9%)	1.97 (1.36–2.85, *p* < .001)	0.99 (0.53–1.85, *p* = .981)

Univariable and multivariable logistic regression analyses revealed that advanced age, elevated red blood cell distribution width (rdw), increased total bilirubin (tbil), elevated aspartate aminotransferase (ast), severe Logistic Organ Dysfunction System score (lods >= 10), and KDIGO stage 3 acute kidney injury (aki_kdigo_stage=3) were all independent risk factors. Concurrently, a higher peripheral oxygen saturation (spo2) and the administration of magnesium_supplementation emerged as significant independent protective factors.

Notably, after adjusting for multiple confounders, several indicators that demonstrated significant differences in the univariable analysis, including white blood cell count (white_blood_cell), blood urea nitrogen (bun), Sequential Organ Failure Assessment score (sofa_score), Simplified Acute Physiology Score II (sapsii_score), Charlson Comorbidity Index (charlson_comorbidity_index), alcoholic etiology (alcoholic_etiology), and the use of vasoactive agents (vasoactive_use), no longer exerted an independent, statistically significant impact on prognosis (*p* > 0.05). Furthermore, patient race and KDIGO stages 1 and 2 acute kidney injury did not demonstrate independent statistical associations with prognosis, as their relationships were significantly influenced by other confounding factors.

### Feature extraction

Figure 4 illustrates the intersection of features selected by different algorithms, as well as the importance ranking of the final core feature set. In this study, three algorithms, such as LASSO, MRMR, and Boruta were employed for feature selection, and the detailed selection process for each algorithm is presented in Supplementary Figure 2.

**Figure 4:**
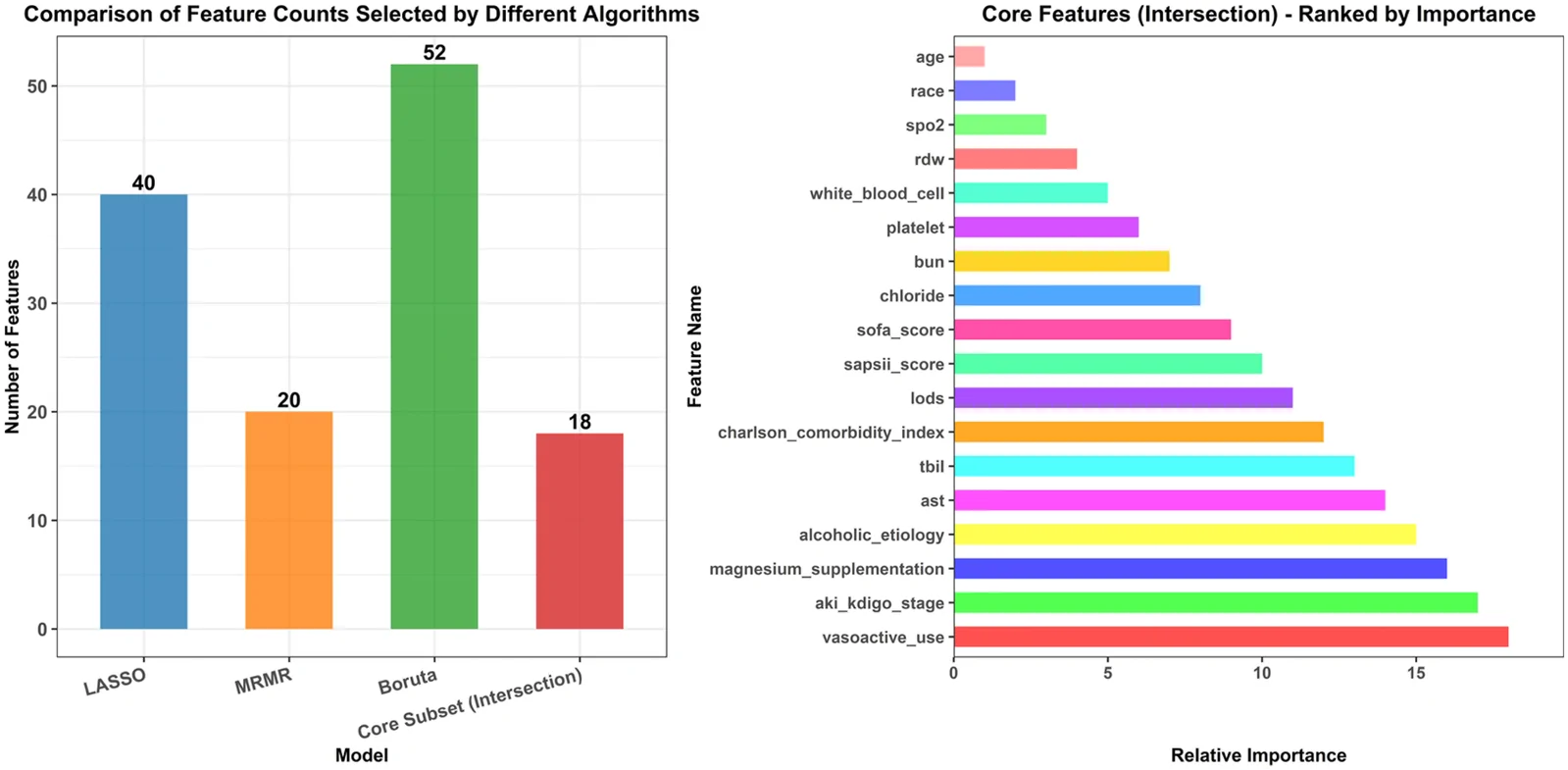
Optimal feature selection process for the merging of three algorithms.

The bar chart on the left demonstrates that the LASSO algorithm selected 40 features, the MRMR algorithm selected 20 features, and the Boruta algorithm selected 52 features. By taking the intersection of the results from the three algorithms, a final set of 18 core features was obtained and utilized as input variables for subsequent machine learning models.

These 18 core features specifically are: age, race, spo2, rdw, white_blood_cell, platelet, bun, chloride, sofa_score, sapsii_score, lods, charlson_comorbidity_index, tbil, ast, alcoholic_etiology, magnesium_supplementation, aki_kdigo_stage, and vasoactive_use.

Overall, this core feature set encompasses multiple dimensions, including organ function, laboratory indicators, etiological metrics, comorbidities, and clinical interventions. The significantly varying contributions of each feature to the model's efficacy laid a solid foundation for constructing a streamlined and efficient machine learning predictive model.

### Model selection and construction

Based on the 18 core features collectively selected by the Boruta, LASSO, and MRMR algorithms, 24 machine learning models were constructed to predict patient clinical outcomes in this study. After a comprehensive comparison of each model's accuracy, AUC, Kappa coefficient, sensitivity, specificity, positive predictive value, negative predictive value, precision, recall, and F1 score (Table 2), combined with Brier scores and DeLong test results (Supplementary Tables 2, 3), it was found that the RF model exhibited the optimal holistic performance.

**Table 2:** Various performance metrics of 24 machine learning models.

Model	AUC_SD	AUC	Accuracy	Kappa	Sensitivity	Specificity	PPV	NPV	Precision	Recall	F1
Random Forest	0.0133	0.8963 (0.8479, 0.9447)	0.8651 (0.8140, 0.9071)	0.6141 (0.4808, 0.7314)	0.7234 (0.5946, 0.8409)	0.9048 (0.8563, 0.9464)	0.6800 (0.5526, 0.8085)	0.9212 (0.8788, 0.9576)	0.6800 (0.5526, 0.8085)	0.7234 (0.5946, 0.8409)	0.7010 (0.5915, 0.7963)
eXtreme Gradient Boosting	0.0103	0.8867 (0.8414, 0.9321)	0.8512 (0.8045, 0.8977)	0.5709 (0.4301, 0.6999)	0.6809 (0.5365, 0.8095)	0.8988 (0.8512, 0.9422)	0.6531 (0.5192, 0.7907)	0.9096 (0.8639, 0.9500)	0.6531 (0.5192, 0.7907)	0.6809 (0.5365, 0.8095)	0.6667 (0.5476, 0.7692)
Bagging Decision Trees	0.0171	0.8713 (0.8151, 0.9274)	0.8512 (0.8047, 0.8977)	0.5709 (0.4288, 0.6996)	0.6809 (0.5365, 0.8158)	0.8988 (0.8535, 0.9464)	0.6531 (0.5200, 0.7907)	0.9096 (0.8625, 0.9497)	0.6531 (0.5200, 0.7907)	0.6809 (0.5365, 0.8158)	0.6667 (0.5455, 0.7709)
LightGBM	0.0162	0.8905 (0.8440, 0.9369)	0.8465 (0.7953, 0.8930)	0.5608 (0.4172, 0.6892)	0.6809 (0.5357, 0.8125)	0.8929 (0.8443, 0.9401)	0.6400 (0.5098, 0.7778)	0.9091 (0.8616, 0.9512)	0.6400 (0.5098, 0.7778)	0.6809 (0.5357, 0.8125)	0.6598 (0.5361, 0.7619)
Gradient Boosting Machine	0.0153	0.8874 (0.8382, 0.9366)	0.8419 (0.7952, 0.8884)	0.5371 (0.3925, 0.6689)	0.6383 (0.5000, 0.7728)	0.8988 (0.8483, 0.9412)	0.6383 (0.5000, 0.7778)	0.8988 (0.8503, 0.9422)	0.6383 (0.5000, 0.7778)	0.6383 (0.5000, 0.7728)	0.6383 (0.5116, 0.7423)
Ridge Regression	0.0243	0.8879 (0.8382, 0.9376)	0.8372 (0.7859, 0.8837)	0.5542 (0.4191, 0.6825)	0.7234 (0.5848, 0.8379)	0.8690 (0.8114, 0.9187)	0.6071 (0.4762, 0.7501)	0.9182 (0.8733, 0.9565)	0.6071 (0.4762, 0.7501)	0.7234 (0.5848, 0.8379)	0.6602 (0.5455, 0.7627)
Linear Discriminant Analysis	0.0278	0.8817 (0.8283, 0.9351)	0.8372 (0.7859, 0.8837)	0.5411 (0.3985, 0.6743)	0.6809 (0.5319, 0.8039)	0.8810 (0.8253, 0.9282)	0.6154 (0.4833, 0.7593)	0.9080 (0.8608, 0.9464)	0.6154 (0.4833, 0.7593)	0.6809 (0.5319, 0.8039)	0.6465 (0.5263, 0.7525)
Bayesian GLM	0.024	0.8841 (0.8318, 0.9364)	0.8326 (0.7767, 0.8837)	0.5314 (0.3829, 0.6638)	0.6809 (0.5454, 0.8043)	0.8750 (0.8203, 0.9235)	0.6038 (0.4694, 0.7501)	0.9074 (0.8590, 0.9477)	0.6038 (0.4694, 0.7501)	0.6809 (0.5454, 0.8043)	0.6400 (0.5176, 0.7500)
Generalized Linear Model	0.0242	0.8834 (0.8307, 0.9360)	0.8326 (0.7767, 0.8837)	0.5314 (0.3829, 0.6638)	0.6809 (0.5454, 0.8043)	0.8750 (0.8203, 0.9235)	0.6038 (0.4694, 0.7501)	0.9074 (0.8590, 0.9477)	0.6038 (0.4694, 0.7501)	0.6809 (0.5454, 0.8043)	0.6400 (0.5176, 0.7500)
C5.0 decision tree algorithm	0.0134	0.8821 (0.8331, 0.9311)	0.8326 (0.7814, 0.8792)	0.5099 (0.3657, 0.6461)	0.6170 (0.4716, 0.7501)	0.8929 (0.8448, 0.9383)	0.6170 (0.4800, 0.7647)	0.8929 (0.8434, 0.9368)	0.6170 (0.4800, 0.7647)	0.6170 (0.4716, 0.7501)	0.6170 (0.4935, 0.7308)
Partial Least Squares DA	0.0276	0.8741 (0.8203, 0.9279)	0.8326 (0.7814, 0.8791)	0.5382 (0.3826, 0.6571)	0.7021 (0.5625, 0.8164)	0.8690 (0.8129, 0.9182)	0.6000 (0.4629, 0.7358)	0.9125 (0.8671, 0.9503)	0.6000 (0.4629, 0.7358)	0.7021 (0.5625, 0.8164)	0.6471 (0.5154, 0.7477)
Quadratic Discriminant Analysis	0.0363	0.7442 (0.6566, 0.8318)	0.8326 (0.7767, 0.8792)	0.4315 (0.2695, 0.5827)	0.4255 (0.2800, 0.5600)	0.9464 (0.9080, 0.9766)	0.6897 (0.5200, 0.8571)	0.8548 (0.8000, 0.9032)	0.6897 (0.5200, 0.8571)	0.4255 (0.2800, 0.5600)	0.5263 (0.3714, 0.6563)
Elastic Net	0.0242	0.8854 (0.8335, 0.9372)	0.8279 (0.7721, 0.8791)	0.5219 (0.3713, 0.6573)	0.6809 (0.5454, 0.8043)	0.8690 (0.8114, 0.9186)	0.5926 (0.4600, 0.7451)	0.9068 (0.8581, 0.9474)	0.5926 (0.4600, 0.7451)	0.6809 (0.5454, 0.8043)	0.6337 (0.5128, 0.7434)
LASSO Regression	0.0242	0.8853 (0.8333, 0.9372)	0.8279 (0.7721, 0.8791)	0.5219 (0.3713, 0.6573)	0.6809 (0.5454, 0.8043)	0.8690 (0.8114, 0.9186)	0.5926 (0.4600, 0.7451)	0.9068 (0.8581, 0.9474)	0.5926 (0.4600, 0.7451)	0.6809 (0.5454, 0.8043)	0.6337 (0.5128, 0.7434)
Support Vector Machine	0.025	0.8827 (0.8298, 0.9356)	0.8279 (0.7721, 0.8791)	0.5219 (0.3738, 0.6516)	0.6809 (0.5365, 0.8000)	0.8690 (0.8118, 0.9211)	0.5926 (0.4583, 0.7415)	0.9068 (0.8589, 0.9474)	0.5926 (0.4583, 0.7415)	0.6809 (0.5365, 0.8000)	0.6337 (0.5116, 0.7402)
Conditional Inference Tree	0.0263	0.8208 (0.7515, 0.8901)	0.8279 (0.7721, 0.8744)	0.5288 (0.3836, 0.6468)	0.7021 (0.5641, 0.8276)	0.8631 (0.8072, 0.9157)	0.5893 (0.4626, 0.7207)	0.9119 (0.8662, 0.9521)	0.5893 (0.4626, 0.7207)	0.7021 (0.5641, 0.8276)	0.6408 (0.5217, 0.7360)
Neural Network	0.0304	0.8369 (0.7728, 0.9009)	0.8140 (0.7581, 0.8651)	0.5400 (0.4120, 0.6630)	0.8298 (0.7174, 0.9286)	0.8095 (0.7456, 0.8683)	0.5493 (0.4347, 0.6765)	0.9444 (0.9048, 0.9786)	0.5493 (0.4347, 0.6765)	0.8298 (0.7174, 0.9286)	0.6610 (0.5565, 0.7591)
Deep Neural Network	0.0238	0.8733 (0.8219, 0.9247)	0.8093 (0.7488, 0.8605)	0.4993 (0.3625, 0.6345)	0.7234 (0.5813, 0.8388)	0.8333 (0.7688, 0.8909)	0.5484 (0.4242, 0.6826)	0.9150 (0.8658, 0.9540)	0.5484 (0.4242, 0.6826)	0.7234 (0.5813, 0.8388)	0.6239 (0.5102, 0.7314)
AdaBoost	0.0145	0.8651 (0.8056, 0.9247)	0.8093 (0.7488, 0.8605)	0.4543 (0.2994, 0.5874)	0.5957 (0.4524, 0.7298)	0.8690 (0.8155, 0.9193)	0.5600 (0.4200, 0.7073)	0.8848 (0.8323, 0.9313)	0.5600 (0.4200, 0.7073)	0.5957 (0.4524, 0.7298)	0.5773 (0.4494, 0.6857)
Flexible Discriminant Analysis	0.0162	0.8576 (0.8018, 0.9135)	0.8000 (0.7442, 0.8512)	0.3814 (0.2228, 0.5304)	0.4681 (0.3243, 0.6087)	0.8929 (0.8395, 0.9375)	0.5500 (0.3913, 0.7027)	0.8571 (0.8047, 0.9074)	0.5500 (0.3913, 0.7027)	0.4681 (0.3243, 0.6087)	0.5057 (0.3678, 0.6265)
Classification and Regression Tree	0.0414	0.6859 (0.5992, 0.7726)	0.7953 (0.7395, 0.8465)	0.4010 (0.2503, 0.5352)	0.5319 (0.3809, 0.6744)	0.8690 (0.8208, 0.9172)	0.5319 (0.3922, 0.6757)	0.8690 (0.8144, 0.9177)	0.5319 (0.3922, 0.6757)	0.5319 (0.3809, 0.6744)	0.5319 (0.4039, 0.6445)
Naive Bayes	0.0268	0.8098 (0.7435, 0.8760)	0.7581 (0.6977, 0.8141)	0.3861 (0.2458, 0.5190)	0.6596 (0.5111, 0.7857)	0.7857 (0.7219, 0.8491)	0.4627 (0.3492, 0.5916)	0.8919 (0.8366, 0.9366)	0.4627 (0.3492, 0.5916)	0.6596 (0.5111, 0.7857)	0.5439 (0.4299, 0.6512)
K-Nearest Neighbors	0.0336	0.7567 (0.6870, 0.8264)	0.7070 (0.6465, 0.7674)	0.3237 (0.1983, 0.4497)	0.7021 (0.5556, 0.8246)	0.7083 (0.6416, 0.7736)	0.4024 (0.3049, 0.5135)	0.8947 (0.8359, 0.9412)	0.4024 (0.3049, 0.5135)	0.7021 (0.5556, 0.8246)	0.5116 (0.4060, 0.6154)
Sparse Linear Discriminant Analysis	0.0316	0.8541 (0.7990, 0.9092)	0.2186 (0.1721, 0.2745)	0.0000 (0.0000, 0.0000)	1.0000 (1.0000, 1.0000)	0.0000 (0.0000, 0.0000)	0.2186 (0.1721, 0.2745)	NaN (NA, NA)	0.2186 (0.1721, 0.2745)	1.0000 (1.0000, 1.0000)	0.3588 (0.2937, 0.4308)

The model achieved an AUC value of 0.8963 (0.8479, 0.9447), demonstrating excellent discriminative ability for high-risk patients. Pairwise comparisons using the DeLong test confirmed that the AUC of the Random Forest model was significantly superior to the most majority of comparative models (*P* < 0.05), verifying its core performance advantage from a statistical perspective. Simultaneously, the performance standard deviation of this model was extremely low (SD = 0.0133), indicating strong generalization ability and minimal fluctuation in predictive efficacy across different data subsets (Figures 5–7). The ROC curves in Figure 6 were generated using a smoothing function for visualization purposes, which resulted in the fitted AUC values displayed in the legend. Please refer to Table 2 for the exact empirical AUC values, which serve as the definitive core evaluation metrics for these models.

**Figure 5:**
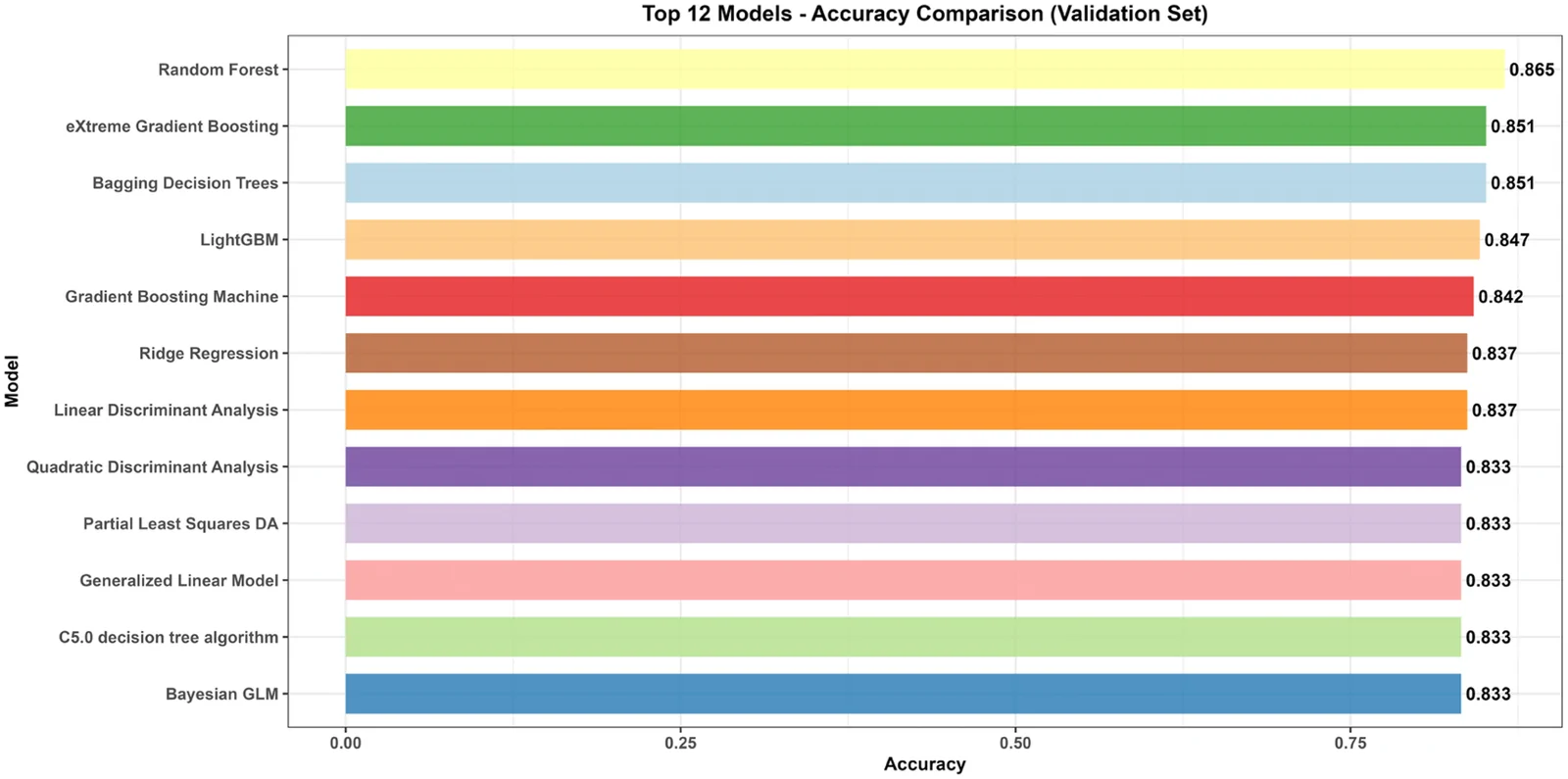
Accuracy of 12 machine learning models.

**Figure 6:**
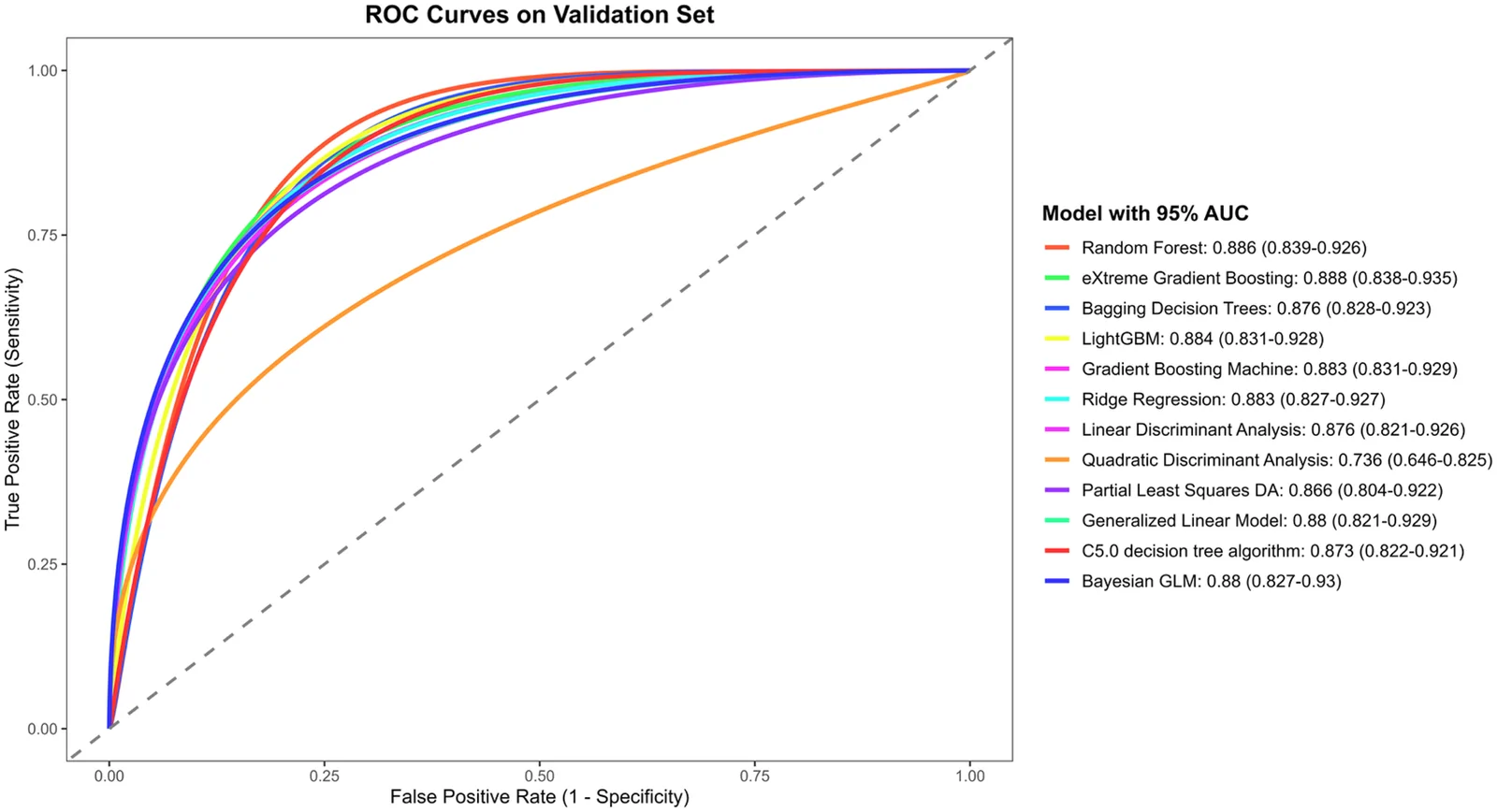
ROC curves on test for 12 machine learning models.

**Figure 7:**
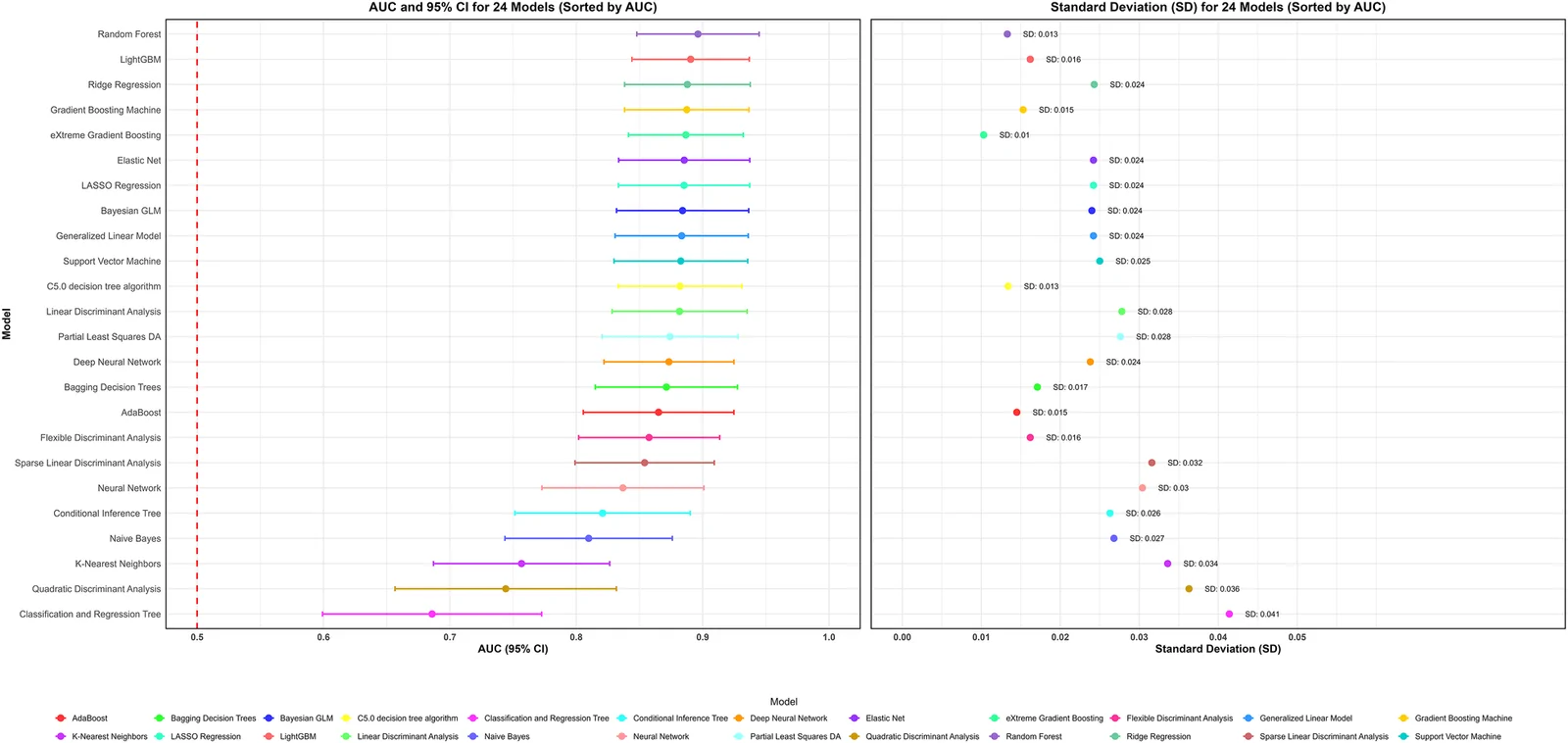
Predictive performance and stability of 24 machine learning models.

These results demonstrate that the Random Forest model is the most stable model for the clinical prediction task in this study, thereby laying a solid foundation for subsequent interpretability analysis of feature contributions using the SHAP method.

Figures 8, 9 comprehensively evaluate the clinical application value of all machine learning models in this study from two core dimensions: first, the degree of agreement between the predicted probability of the event and the actual outcome (i.e., calibration performance); and second, the overall net benefit the model can provide in clinical decision-making.

**Figure 8:**
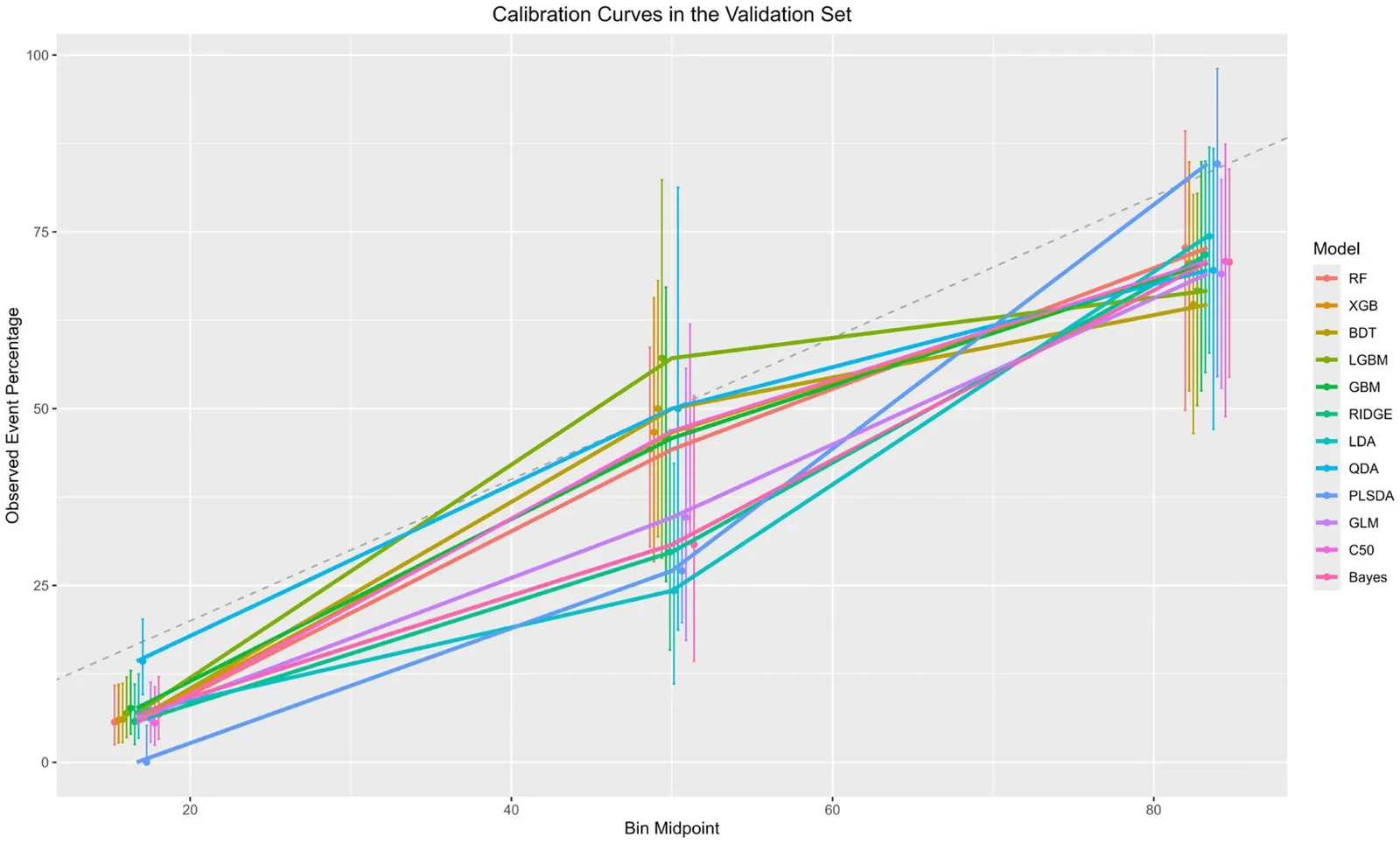
Calibration curves in the validation set.

**Figure 9:**
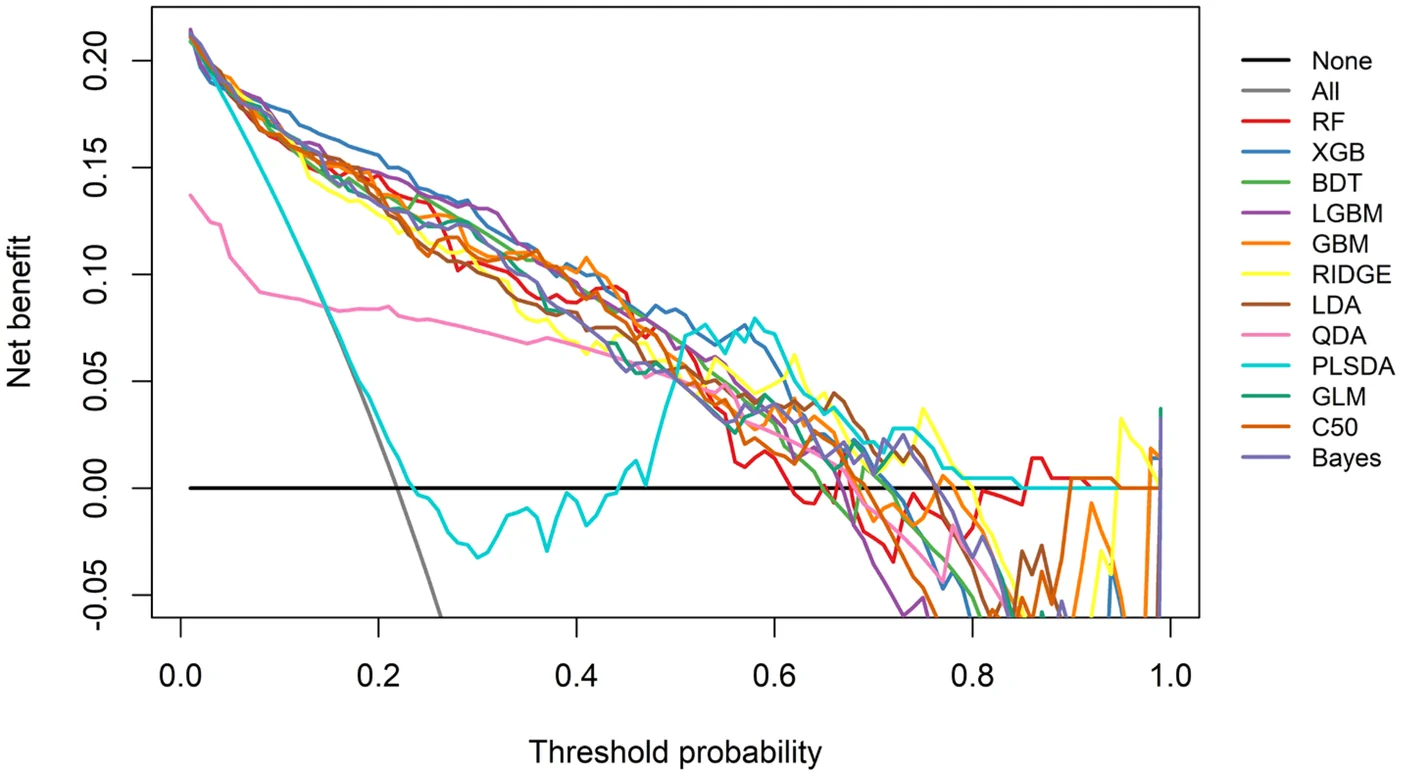
Decision curve analysis plot of the models.

The calibration curve results in Figure 8 indicate a significant divergence in the calibration performance among the machine learning models. Certain models (such as QDA and PLSDA) exhibit substantial curve deviations across different predicted probability intervals, presenting issues of systematically overestimating or underestimating the true risk. In contrast, tree-based ensemble models represented by RF (red line), XGB and LGBM demonstrate excellent calibration. Notably, the RF model curve closely aligns with the dashed diagonal line representing the ideal calibration state; across the most majority of risk intervals, its predicted probability remains highly consistent with the actual outcome incidence. Compared to other models with obvious error intervals, the RF model exhibits exceptional predictive stability and high accuracy in absolute risk assessment.

The DCA in Figure 9 further quantifies the application value of each model in real-world clinical scenarios. In the plot, the horizontal black line (treat none) and the descending gray line (treat all) correspond to the baseline net benefits of two extreme clinical management strategies, respectively. The results demonstrate that within a broad range of clinically applicable risk thresholds (approximately 0.05 to 0.7), the clinical net benefits of ensemble models such as RF, XGB, and LGBM consistently rank in the top tier. Although the net benefits of XGB and LGBM are slightly higher than that of RF at certain specific thresholds (e.g., 0.2 to 0.5), the RF model (red curve) maintains a highly stable and significant positive net benefit across the entire interval, enabling the precise identification of high-risk populations while effectively avoiding unnecessary overtreatment interventions. In comparison, the net benefits of most comparative models, such as QDA and RIDGE, decline rapidly as the risk threshold increases, indicating limited clinical application value.

Considering calibration ability, clinical decision net benefit, and the previously established discriminative efficacy, RF emerges as the model with the optimal holistic performance and the most balanced behavior in this study. This model not only effectively overcomes the probability calibration bias commonly found in most traditional algorithms but also possesses solid potential for clinical utility. Consequently, this establishes a reliable evidence-based foundation for the subsequent interpretability analysis of feature contributions using SHAP, as well as the real-world clinical translation of the model.

### Model interpretation and deployment

In this study, the SHAP method was employed to conduct an interpretability analysis on the optimal Random Forest model. By quantifying the magnitude and direction of each feature's impact on the model's predictions, we addressed the inherent “black-box” issue of machine learning algorithms and validated the clinical rationality of the core predictive indicators, as detailed in Figures 10, 11.

**Figure 10:**
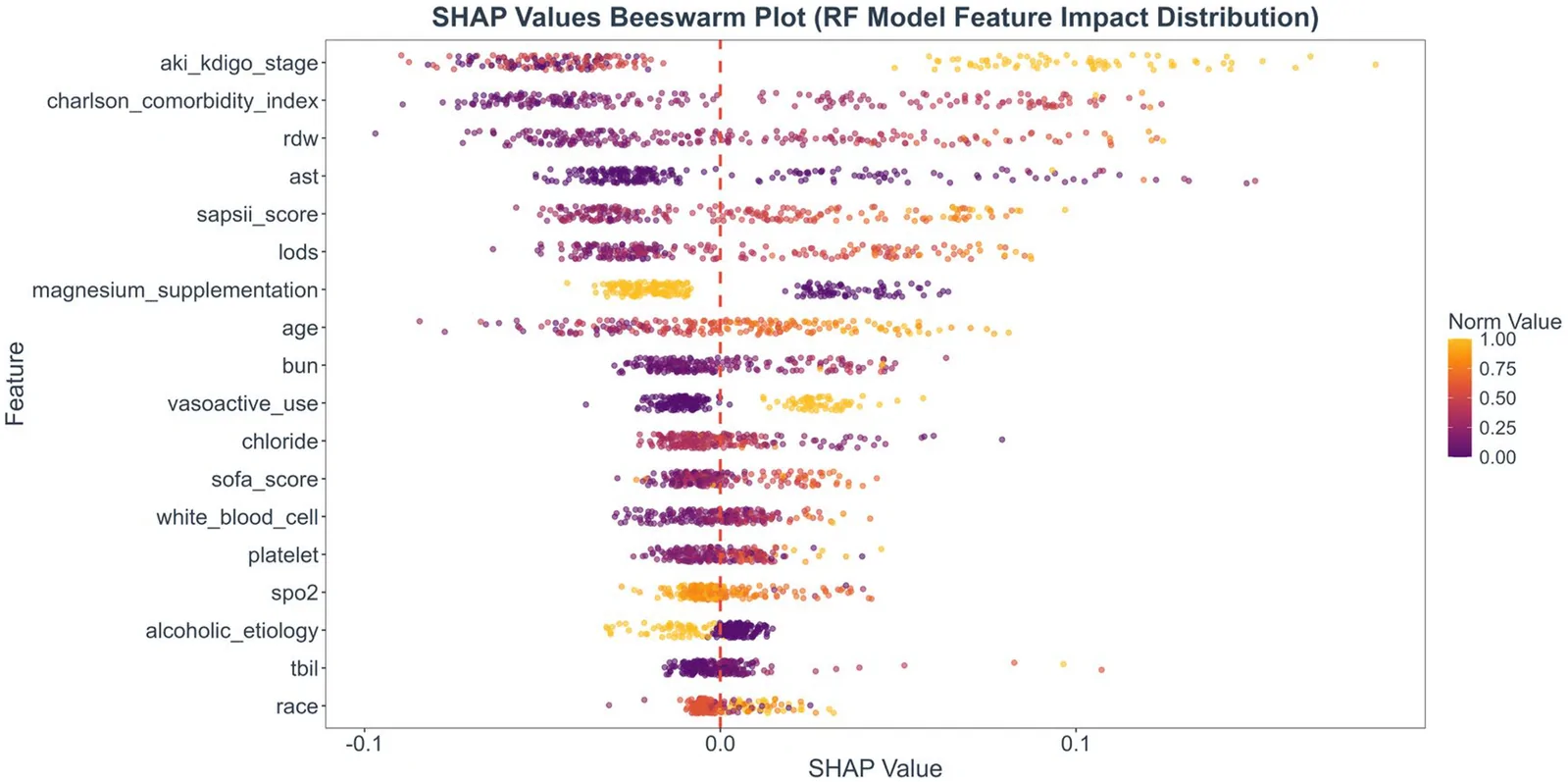
Beeswarm plot of SHAP values based on random forest model.

**Figure 11:**
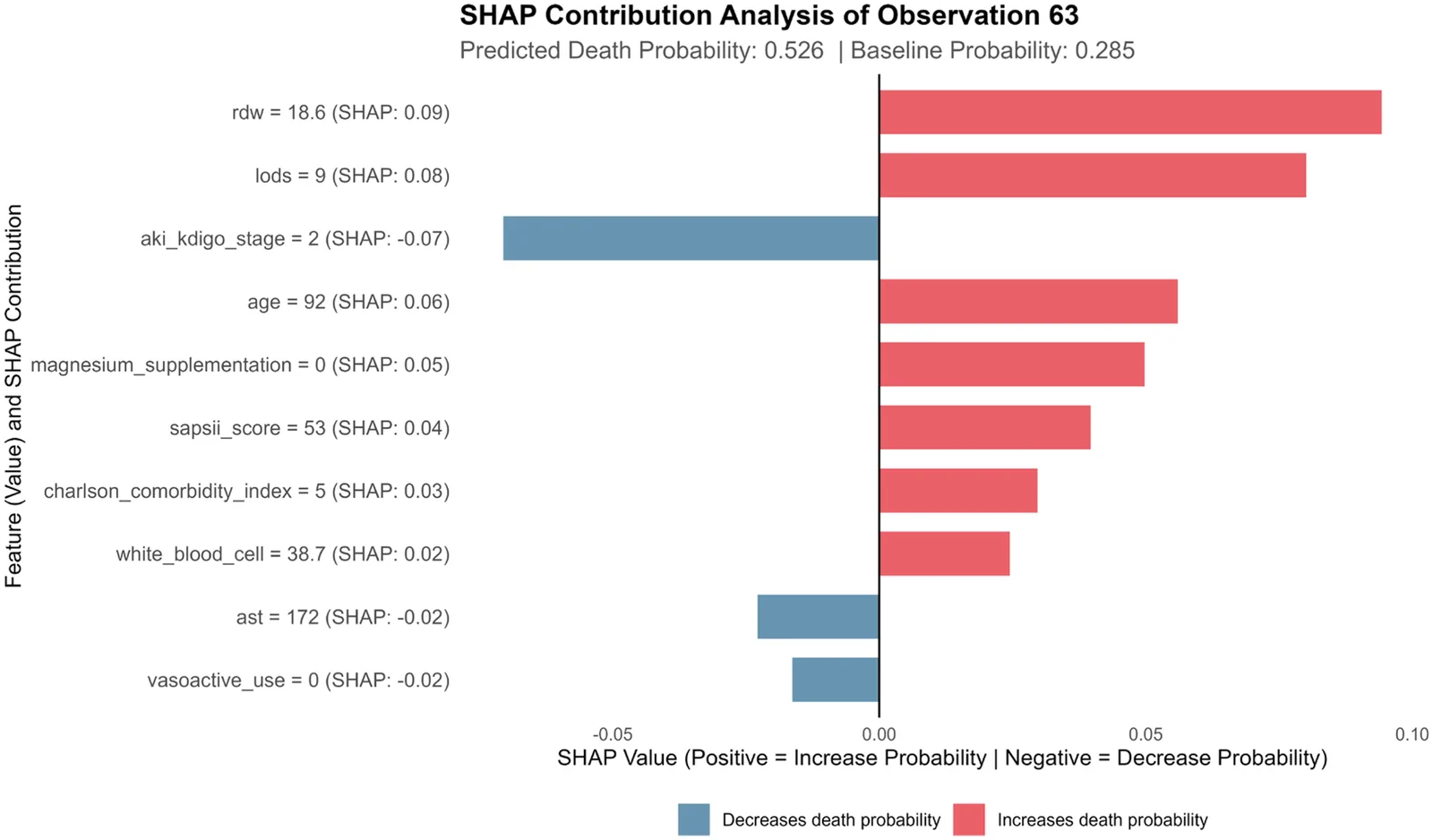
SHAP feature contribution analysis plot for a single sample.

Regarding the overall effect, the SHAP beeswarm plot in Figure 10 visually illustrates the global impact distribution and the importance ranking of all included features (descending along the *y*-axis). The results indicate that aki_kdigo_stage, charlson_comorbidity_index, rdw, ast, and sapsii_score are the top five crucial indicators driving the predictive performance of the model.

The high values (represented by yellow/orange dots in the plot) of indicators such as aki_kdigo_stage, charlson_comorbidity_index, rdw, ast, and sapsii_score are predominantly distributed to the right of the central reference line, corresponding to positive SHAP values. This demonstrates that abnormal elevations or aggravated clinical scores in these indicators significantly increase the risk of adverse outcomes, such as mortality.

Conversely, the high values (yellow/orange dots) of certain features are mostly distributed within the negative SHAP value range (left of the midline). For instance, patients receiving magnesium_supplementation or presenting with an alcoholic_etiology correspond to negative model contributions, suggesting that these two features exert a pronounced protective effect within the model. Similarly, elevated spo2 levels also map to negative SHAP values, exerting a protective effect by reducing the risk of death.

From the perspective of single-instance prediction, the SHAP contribution plot (bar chart) in Figure 11 intuitively illustrates the underlying logic of the model in conducting individualized risk assessments. For this specific patient, the model predicted a mortality probability of 0.526, which is notably higher than the baseline probability of 0.285. The identification of this patient as a high-risk individual is the result of the combined positive and negative effects of multiple clinical features.

Specifically, a markedly elevated red blood cell distribution width (rdw=18.6, SHAP value = + 0.09), severe organ dysfunction (lods=9, SHAP value = + 0.08), extreme advanced age (age=92, SHAP value = + 0.06), and the absence of magnesium supplementation (magnesium_supplementation=0, SHAP value = + 0.05) served as the primary positive driving factors that elevated the patient's mortality risk. Concurrently, the patient's acute kidney injury being at stage 2 (aki_kdigo_stage=2, SHAP value = −0.07), a relatively lower transaminase level (ast = 172, SHAP value = −0.02), and the non-use of vasoactive agents (vasoactive_use = 0, SHAP value=−0.02) acted as negative contributors, slightly offsetting the overall risk escalation.

This individualized analysis thoroughly demonstrates that the Random Forest model can synthesize multi-dimensional clinical indicators to generate highly interpretable and targeted individualized risk assessments for critically ill patients. By elucidating the clinical value and mechanistic role of the core predictive indicators from both global and local (single-instance) perspectives, this SHAP interpretability analysis substantially enhances the transparency and clinical credibility of the model, establishing a solid evidence-based foundation for its future clinical translation and practical application.

Figure 12 displays the visualization of SHAP dependence relationships for multiple features, systematically demonstrating the non-linear associations between the predicted mortality risk and six core features: aki_kdigo_stage, charlson_comorbidity_index, rdw, ast, sapsii_score, and lods. The red fitting trend lines in the plots intuitively quantify the dynamic impact of numerical variations within a single feature on its predictive contribution.

**Figure 12:**
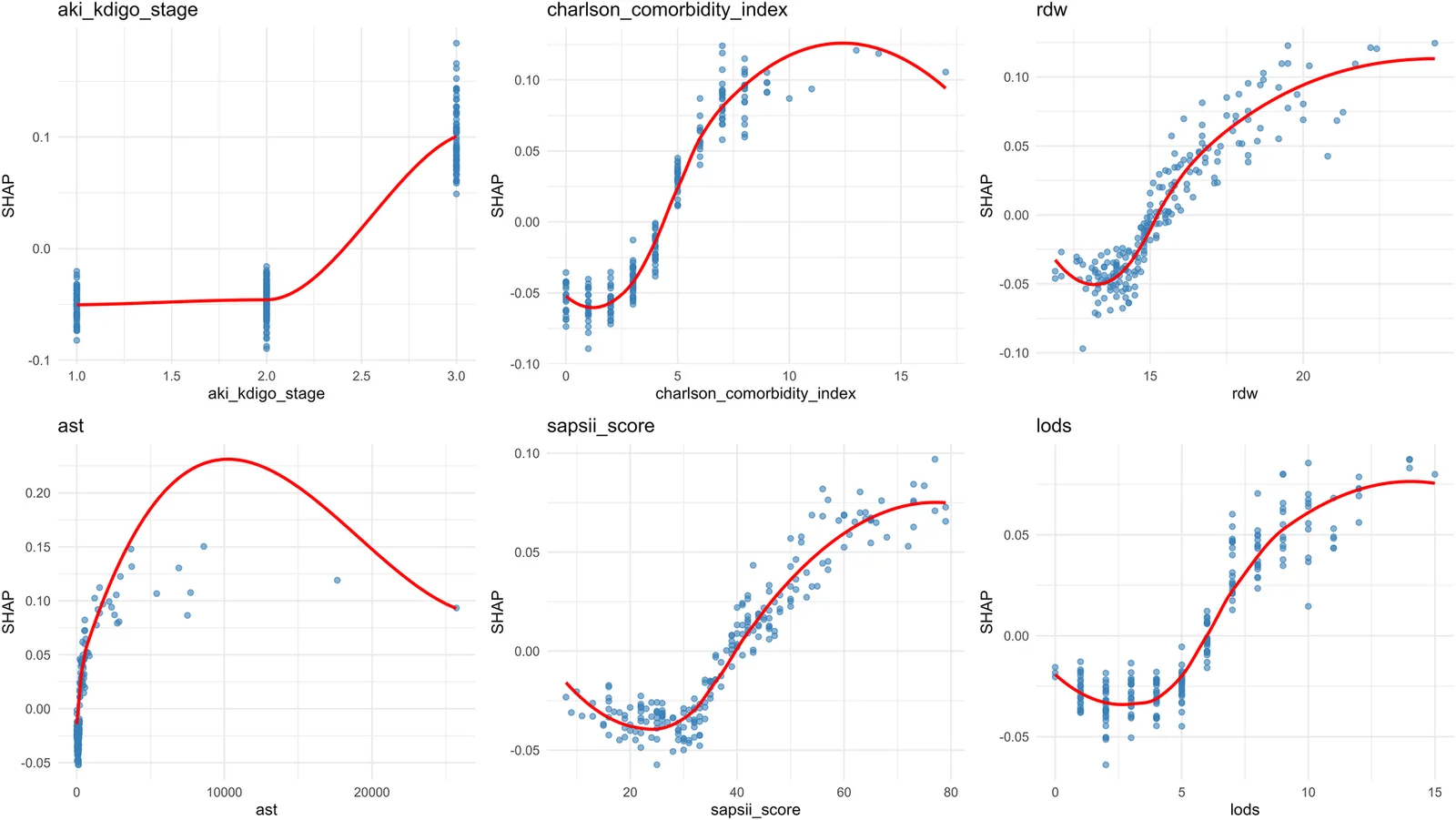
Visualization of SHAP dependence relationships for multiple features.

Overall, aki_kdigo_stage exhibits a pronounced non-linear abrupt effect: when patients are at stages 1 and 2, the SHAP values remain in the negative range below the zero line (indicating a relatively low risk); however, once the kidney injury deteriorates to stage 3, the mortality-promoting risk intensifies sharply, with the SHAP value leaping into a significantly positive high-level position.

Meanwhile, charlson_comorbidity_index, rdw, sapsii_score, and lods all present a typical “S-shaped” threshold and saturation effect. At lower scores or levels (e.g., rdw < 14, sapsii_score < 35, lods < 5), they correspond to negative SHAP values, suggesting a lower risk of mortality. Once crossing their specific danger thresholds, their mortality-promoting effects accelerate sharply, with SHAP values climbing almost linearly as the values increase. When the indicators reach extremely high intervals, this risk-promoting effect gradually saturates and enters a plateau phase.

Notably, ast displays a distinct non-linear trajectory characterized by an initial rapid escalation followed by a high-level slight decline: upon the initial abnormal elevation of its value, the predicted risk climbs rapidly to a peak; however, after reaching an extremely elevated abnormal range, the marginal growth of its risk-promoting effect flattens or even exhibits a minor attenuation.

The findings in this section are highly consistent with the global SHAP analysis, further elucidating the dynamic and non-linear mechanisms through which these six core clinical indicators influence patient prognosis with fine-grained precision.

We integrated the final RF model into an interactive Shiny-based web application that provides individualized survival predictions and interpretations, as well as global model interpretation, as shown in Figure 13. This web application is accessible via the URL: https://medicalpredictor.shinyapps.io/Online_Prediction_of_60-Day_Survival_Status_in_Patients.

**Figure 13:**
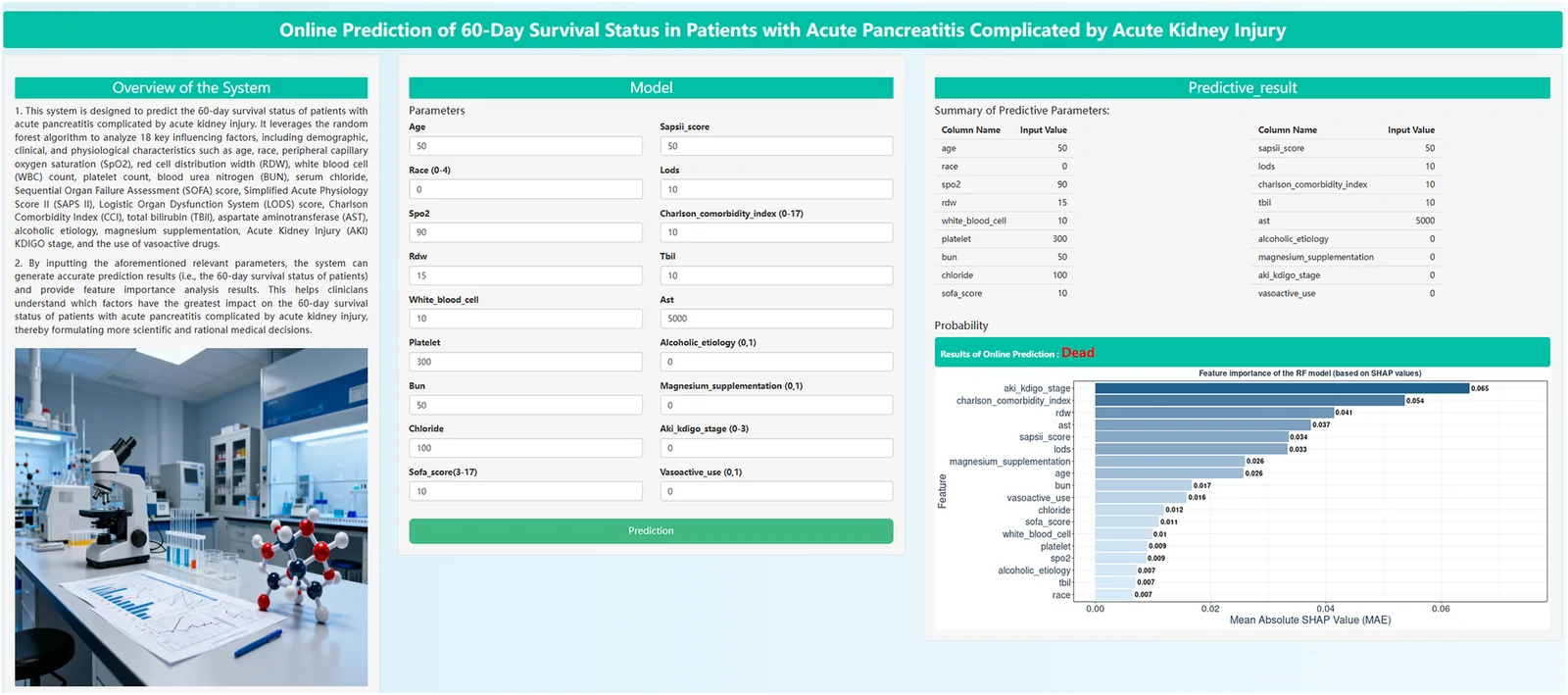
Online prediction system interface for survival status in patients with acute pancreatitis complicated by acute kidney injury.

Focusing on the high-risk clinical scenario of acute pancreatitis complicated by acute kidney injury, this system takes the Random Forest algorithm as its core and constructs an integrated workflow encompassing parameter entry, one-click prediction, and result visualization. It can not only accurately output the survival probability of patients but also enable clinicians to intuitively understand the key influencing factors underlying the predictions through SHAP feature importance analysis. This not only improves the efficiency of clinical decision-making but also enhances the interpretability and credibility of the model, thereby providing strong support for the formulation of personalized treatment plans. Importantly, the online prediction tool developed in this study is intended strictly for research and educational purposes. It is designed to serve as an adjunct to clinical decision-making and must not be used as a replacement for the professional judgment of healthcare providers.

### Model external validation

To rigorously evaluate the generalizability and robustness of the developed machine learning models, an external validation was conducted utilizing the independent eICU Collaborative Research Database. To ensure methodological consistency, the variable screening and mapping rules for the eICU cohort were kept strictly identical to those established for the MIMIC-IV training set. All 18 core predictors (including age, sofa_score, aki_kdigo_stage, and vasoactive_use) were successfully acquired and aligned between the two databases. After applying synchronized inclusion and exclusion criteria, the final external validation cohort comprised 153 patients, consisting of 143 survivors and 10 mortality cases. This composition presents an extreme class imbalance, with a baseline mortality rate of approximately 6.5%. The comprehensive baseline clinical and demographic characteristics of this external cohort are detailed in Supplementary Table 4.

ROC analysis (Figure 14B) demonstrated that the optimal RF algorithm maintained a reliable overall discriminative efficacy, achieving an AUC of 0.8122 (95% CI: 0.7139–0.9106). The external validation curve deviated significantly from the reference diagonal line representing random guessing, confirming the reliable risk stratification capability of the model in an independent population. At the optimal classification threshold (Figure 14A), the model maintained a clinical sensitivity of 0.70. Driven directly by the mathematical effects of extreme class imbalance and cross-center data heterogeneity, certain classification metrics experienced an expected decline, with the precision falling to 0.18 and the F1-score dropping to 0.29. Furthermore, the calibration curve (Figure 14C) indicated a certain degree of probability overestimation, which is highly typical when transferring models to an external cohort with a lower baseline incidence. Nonetheless, DCA (Figure 14D) confirmed that the model still provided a positive clinical net benefit within lower threshold probability intervals (approximately 0% to 25%), demonstrating its clinical utility as a robust early-warning screening tool for high-risk AP-AKI patients in a multi-center setting.

**Figure 14:**
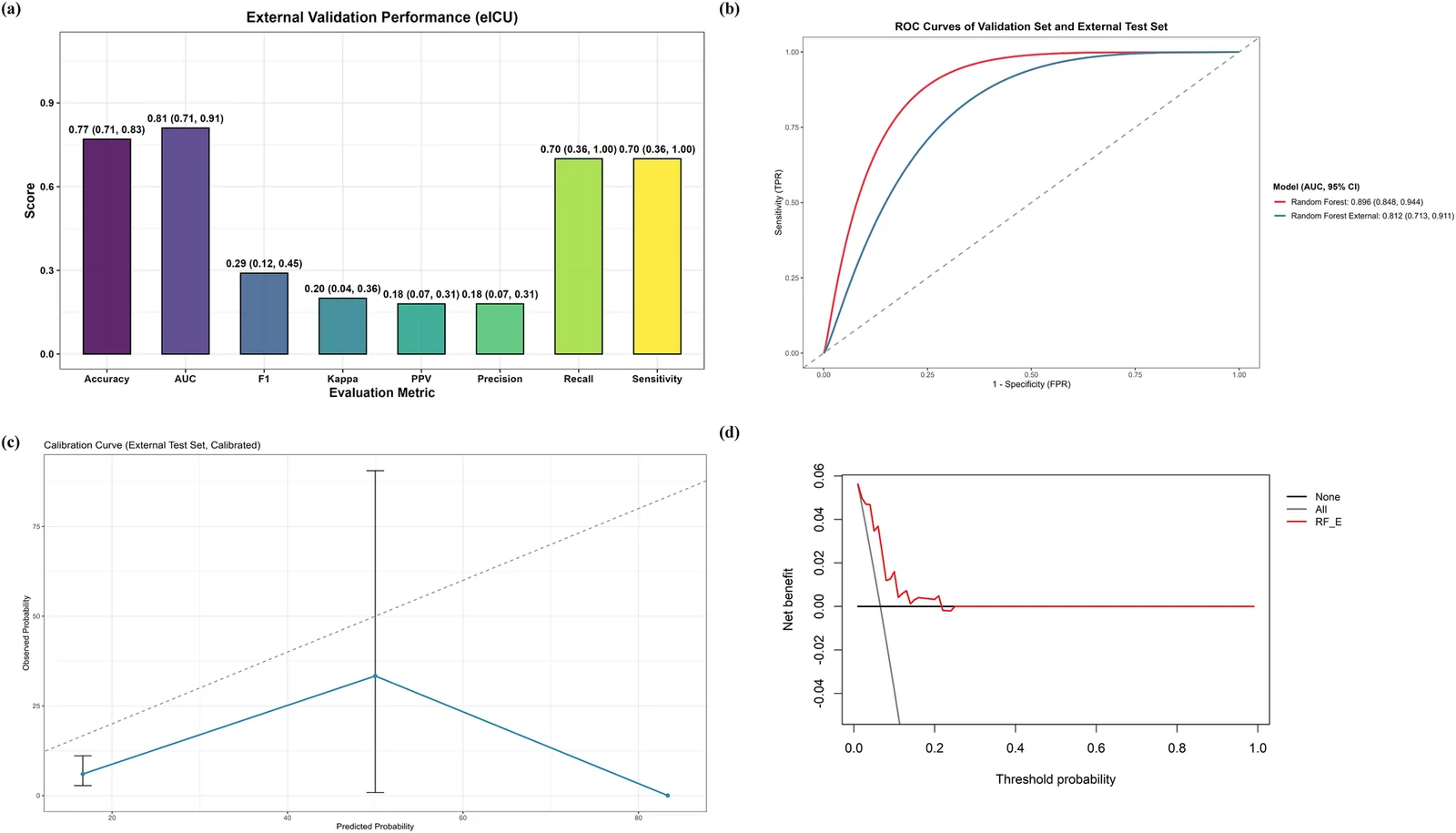
Comprehensive external validation results of the RF model (eICU dataset). **(a)** Model evaluation metrics; **(b)** ROC curves; **(c)** Calibration curve; **(d)** DCA curve.

## Discussion

To comprehensively and objectively analyze the complex clinical phenotypes and the holistic status of diagnosis and treatment in patients with acute pancreatitis complicated by acute kidney injury, this study further expanded the clinical variable repository. A systematic baseline analysis was conducted focusing on the etiology of pancreatitis, intensive care unit interventions, nutritional support regimens, and the dynamic trends of core laboratory indicators. Incorporating these high-dimensional clinical data not only delineates the critical care characteristics of the study population with high granularity but also provides a complete clinical background for exploring the potential impacts of different intervention strategies on patient prognosis.

Notably, a higher proportion of patients in the non-survivor group received advanced life support measures, such as renal replacement therapy and vasoactive agents, indicating a more critical degree of organ failure in this group. However, when constructing the machine learning models, to circumvent issues related to data leakage and treatment paradoxes, these intervention-based variables and long-term dynamic indicators that evolve with disease progression were utilized exclusively for baseline clinical description and were not incorporated into the feature set of the early risk prediction model. Combining the comprehensive clinical baseline data, this study ultimately included 721 patients with AP-AKI followed for 60 days, among whom 562 survived and 159 died.

In this study, univariable and multivariable logistic regression analyses were performed to systematically screen independent factors associated with patients. The final core clinical indicators selected were highly consistent with the feature importance ranking derived from the subsequent SHAP interpretability analysis, establishing a solid evidence-based foundation for constructing the machine learning predictive models (29).

The multivariable logistic regression results indicated that advanced age was an independent risk factor in AP-AKI patients (OR = 1.03, 95% CI: 1.00–1.05, *P* = 0.020). The mean age at admission in the non-survivor group was 70.4 ± 15.3 years, which was significantly higher than that in the survivor group (59.6 ± 18.0). Elderly patients suffer from a marked decline in systemic organ reserve capacity and a diminished tolerance to the systemic inflammatory response syndrome triggered by acute pancreatitis; thus, mortality risk escalates significantly with advancing age. Regarding the assessment of organ dysfunction, a severe Logistic Organ Dysfunction System score (lods >= 10) was confirmed as a core risk factor (OR = 3.34, 95% CI: 1.00–11.11, *P* = 0.050), suggesting that systemic injury and sequential organ failure secondary to acute pancreatitis are critical determinants of patient prognosis (30). In addition, although the charlson_comorbidity_index indicated an increased risk in the univariable analysis, it did not demonstrate independent statistical significance after multivariable adjustment (*P* > 0.05).

As an indicator directly reflecting target organ damage in the kidneys, KDIGO stage 3 acute kidney injury (aki_kdigo_stage = 3) exerted a particularly prominent risk-promoting effect on mortality. Compared to patients with no or mild kidney injury stages, patients at KDIGO stage 3 faced an approximately four-fold increase in mortality risk (OR = 4.02, 95% CI: 1.91–8.44, *P* < 0.001). This demonstrates that rapid and severe impairment of renal function triggers severe fluid retention, electrolyte imbalances, and toxin accumulation, representing the most critical clinical event driving mortality outcomes.

Liver injury biomarkers and hematological abnormalities also serve as important indicators of poor prognosis in AP-AKI patients. Multivariable analysis showed that elevated tbil (OR = 1.09, 95% CI: 1.04–1.15, *P* = 0.001) and a substantial rise in ast (*P* = 0.019) both significantly increased the risk of death; the mean ast level in the non-survivor group reached as high as 1450.6 ± 3736.9 U/L, far exceeding that of the survivor group (371.5 ± 1334.1) (31). This suggests that hepatocellular injury and cholestasis induced by pancreatic inflammation can create a vicious cycle with renal impairment, further aggravating systemic metabolic disturbances. Furthermore, an elevated rdw also possessed high predictive value (OR = 1.33, 95% CI: 1.20–1.48, *P* < 0.001), with a mean value of 16.8 ± 2.8% in the non-survivor group, indicating the presence of intense systemic inflammation, oxidative stress, and hematological dysfunction in these critically ill patients.

This study also identified several independent protective factors of substantial clinical significance. A higher spo2 was associated with a significantly reduced risk of mortality (OR = 0.89, 95% CI: 0.81–0.98, *P* = 0.022), suggesting that maintaining adequate tissue oxygenation and initiating early respiratory support during the acute phase are of paramount importance. Regarding therapeutic interventions, the administration of magnesium supplementation exerted an extremely prominent protective effect on prognosis (OR = 0.41, 95% CI: 0.25–0.70, *P* < 0.001). Severe acute pancreatitis frequently causes profound hypomagnesemia, and magnesium plays a critical role in stabilizing endothelial function and modulating inflammation. Consequently, timely magnesium supplementation not only corrects electrolyte derangements but also mitigates systemic inflammation and microcirculatory dysfunction, thereby protecting against further organ damage such as acute kidney injury. This underscores the clinical value of early electrolyte intervention in this high-risk population. It is worth noting that features such as alcoholic_etiology did not exert a significant independent impact on long-term prognosis after multivariable adjustment.

The multi-feature SHAP dependence analysis in Figure 12 comprehensively elucidates the non-linear mechanistic roles of six core predictors (aki_kdigo_stage, charlson_comorbidity_index, rdw, ast, sapsii_score, and lods) on mortality risk from the perspective of dynamic numerical changes. This effectively addresses the limitation of global feature importance analysis, which fails to reflect the dynamic variations of indicator effects.

Based on their patterns of impact on the outcome, the six core indicators in Figure 12 can be classified into three distinct categories:

The first category consists of non-linear abrupt and step-wise risk-promoting indicators. The representative feature is aki_kdigo_stage. This indicator demonstrates a pronounced abrupt jump effect: when kidney injury is at stages 1 and 2, the SHAP values remain within the negative range below the zero line, representing a relatively low-risk state; however, once the kidney injury progresses to stage 3, the SHAP value exhibits a sharp, step-wise positive leap, becoming a core factor driving the predicted mortality risk.

The second category comprises clinical scores and biochemical indicators exhibiting non-linear threshold and saturation effects. This category includes charlson_comorbidity_index, rdw, sapsii_score, and lods. When these four indicators are at normal or relatively low levels, they exhibit a baseline protective effect against mortality risk (negative SHAP values). Once the indicators cross specific abnormal danger thresholds (e.g., rdw around 14, sapsii_score around 35, and lods around 5), their mortality-promoting effects accelerate sharply in a nearly linear fashion. When the values reach extremely high levels, the risk-promoting effect gradually stabilizes and reaches a plateau (saturation effect), which is highly consistent with the pathophysiological characteristics of exhaustive depletion of systemic compensatory capacity in the late stages of organ failure in critically ill patients (32).

The third category consists of biochemical indicators with typical inverted U-shaped biphasic effects. The representative feature is ast. This indicator presents a unique, parabola-like non-linear trajectory: upon initial abnormal elevation, the predicted mortality risk climbs rapidly to a peak; however, when the transaminase level reaches an extremely severe abnormal range (e.g., > 10,000 U/L), the marginal growth of its risk-promoting effect levels off or even exhibits a minor attenuation.

These findings further confirm that the impact of core clinical indicators on the prognosis of critically ill patients is not a simple linear superposition. Accurately identifying the danger thresholds and saturation plateaus of these indicators can provide clear benefits for formulating individualized, stage-specific precision intervention strategies in clinical practice.

In this study, a total of 24 machine learning models were developed, among which the RF model demonstrated the optimal holistic performance, achieving an AUC of 0.8963 (0.8479, 0.9447) in the internal validation set. This model performed excellently across multiple classification evaluation metrics, including accuracy, sensitivity, and specificity. Combined with the results of the calibration curve and DCA, the model not only possesses outstanding discriminative ability for clinical outcomes and high accuracy in absolute risk assessment but also yields a substantial clinical net benefit across a wide range of decision thresholds. Tree-based ensemble models (e.g., RF and GBM) outperformed traditional linear models in our AP-AKI cohort. As evidenced by the S-shaped curves in Figure 12, tree-based algorithms effectively capture non-linear threshold effects of clinical parameters. Furthermore, they naturally resolve complex, higher-order feature interactions without requiring pre-specified terms. These advantages make them exceptionally well-suited for deciphering the complex pathophysiology of severe acute pancreatitis and accurately predicting acute kidney injury.

Furthermore, rigorous external testing was conducted on the optimal Random Forest model utilizing the independent eICU dataset. The results indicated that, despite cross-center data heterogeneity and extreme class imbalance, the model maintained robust generalization efficacy, achieving an external validation AUC of 0.8122 (0.7139, 0.9106). Furthermore, although the extremely low mortality rate (approximately 6.5%) in the eICU validation cohort mathematically limits the PPV to 0.18, the model maintains high sensitivity. In highly imbalanced real-world ICU settings, the model can assist clinicians in excluding low-risk patients.

To facilitate the clinical translation of our research findings, an interactive Shiny web application (https://medicalpredictor.shinyapps.io/Online_Prediction_of_60-Day_Survival_Status_in_Patients) was developed based on the optimal RF model, establishing a complete workflow that integrates clinical indicator entry, prognostic outcome visualization, and risk factor interpretation. Clinicians can conveniently utilize this tool without the need to master complex programming or data analysis skills.

During operation, clinicians only need to input the 18 core indicators selected through feature engineering, prominently encompassing aki_kdigo_stage, organ dysfunction scores (such as sapsii_score and lods), rdw, ast, spo2, magnesium_supplementation, and alcoholic_etiology to rapidly obtain the predicted mortality risk probability for patients with acute pancreatitis complicated by acute kidney injury, while simultaneously viewing individualized risk attribution diagrams generated via the SHAP algorithm.

This online application substantially lowers the barrier to implementing advanced machine learning models in critical care clinical practice, significantly enhancing the practical utility and translational value of the predictive model. It serves as an accessible and intuitive evidence-based decision support tool for early risk stratification, precise high-risk screening, and the formulation of individualized intervention strategies for AP-AKI patients.

Although this study establishes a robust evidence-based foundation and a highly applicable machine learning tool for the prognostic assessment of patients with acute pancreatitis complicated by acute kidney injury, several limitations remain to be addressed in future work.

First, the retrospective design inherently introduces risks of selection and information biases. While our Random Forest model demonstrated robust discrimination in the independent eICU external test set (AUC = 0.8122), it experienced certain limitations in calibration and overall clinical net benefit. This was primarily driven by the extremely low incidence of true mortality events in the external cohort (only 6.5%, 10 out of 153 cases), which inevitably led to statistical fluctuations and an expectedly lower PPV. Furthermore, to alleviate the systematic overestimation of predictive probabilities caused by the SMOTE oversampling technique used in the training set, we implemented an advanced Prior Probability Correction technique during external validation. These phenomena objectively reflect the natural performance of machine learning models when confronted with extreme class imbalance and cross-center baseline prevalence shifts in real-world data. Future large-scale, multi-center, prospective cohort studies are required to further validate and dynamically recalibrate the model's predictive efficacy in complex clinical settings.

Second, regarding the feature selection strategy, the final model incorporated only 18 core clinical baseline indicators obtained early upon admission. This was done to ensure the model functions as a reliable “early-warning” tool and to strictly circumvent data leakage and treatment paradoxes. Consequently, longitudinal follow-up metrics during intensive care, radiomics features, and subsequent advanced life support interventions were not included as predictors, which partially restricts the model's ability to dynamically track the longitudinal trajectory of disease evolution. Incorporating temporal deep learning algorithms to construct dynamic predictive models represents an important direction for future optimization.

Finally, this study remains in the preliminary exploration stage regarding clinical translation and subgroup analyses. Although an online Shiny-based prediction application was deployed, it has yet to undergo rigorous clinical effectiveness validation through prospective randomized controlled trials (RCTs). Furthermore, in a real-world ICU environment, requiring clinicians to manually input 18 features is both time-consuming and prone to human error. Future deployment must focus on direct integration with Electronic Health Record (EHR) systems to enable automated data extraction and real-time risk calculation. Meanwhile, constrained by the overall sample size, we were unable to perform stratified analyses for specific subgroups (e.g., age brackets, etiologies) to explore inter-group heterogeneity. Additionally, while the SHAP analysis enhanced the “white-box” interpretability of the model, it cannot deeply elucidate the complex pathophysiological interactions at the molecular mechanism level. These limitations will serve as the primary focus for the continuous iterative optimization of this AI-assisted clinical decision support system.

## Conclusion

This study developed and validated a highly interpretable Random Forest prognostic model for the early prediction in patients with AP-AKI. Through a systematic evaluation of 24 machine learning models and a rigorous independent external validation utilizing the eICU database, the Random Forest model was demonstrated to achieve the optimal holistic performance in terms of discriminative ability, overall calibration effect, and clinical decision net benefit, while effectively uncovering the non-linear, dynamic associations between complex clinical indicators and patient prognosis.

Combined with multivariable logistic regression and SHAP interpretability analyses, this study ultimately identified 18 core prognostic indicators (prominently encompassing aki_kdigo_stage, organ dysfunction scores, liver injury indicators such as tbil and ast, rdw, and magnesium_supplementation interventions). This not only accurately characterizes the risk stratification profiles of AP-AKI patients but also establishes a solid evidence-based foundation for early clinical interventions.

The accompanying interactive Shiny web application developed in this study provides clinicians with an intuitive and convenient tool for early individualized risk screening and clinical decision support. Future research will prioritize large-scale, multi-center prospective cohort studies and randomized controlled trials (RCTs) to further validate and dynamically recalibrate the practical utility of this tool in real-world clinical settings. (32)

## Data Availability

The original contributions presented in the study are included in the article/Supplementary Material, further inquiries can be directed to the corresponding author.
